# Peptoid Efficacy against Polymicrobial Biofilms Determined by Using Propidium Monoazide‐Modified Quantitative PCR

**DOI:** 10.1002/cbic.201600381

**Published:** 2016-11-30

**Authors:** Yu Luo, Hannah L. Bolt, Gabriela A. Eggimann, Danny F. McAuley, Ronan McMullan, Tanya Curran, Mei Zhou, Professor Colin A. B. Jahoda, Steven L. Cobb, Fionnuala T. Lundy

**Affiliations:** ^1^ Centre for Experimental Medicine The Wellcome–Wolfson Institute for Experimental Medicine School of Medicine Dentistry and Biomedical Sciences Queen's University Belfast 97 Lisburn Road Belfast BT9 7BL UK; ^2^ Durham University Department of Chemistry Biophysical Sciences Institute South Road Durham DH1 3LE UK; ^3^ Regional Virus Laboratory Kelvin Building Royal Victoria Hospital Belfast Health and Social Care Trust Grosvenor Road Belfast BT12 6BA UK; ^4^ School of Pharmacy Queen's University Belfast 97 Lisburn Road Belfast BT9 7BL UK; ^5^ Durham University School for Biological and Biomedical Sciences South Road Durham DH1 3LE UK

**Keywords:** antibacterial, antibiotics, antiproliferation, cross kingdom, peptoid, quantification

## Abstract

Biofilms containing *Candida albicans* are responsible for a wide variety of clinical infections. The protective effects of the biofilm matrix, the low metabolic activity of microorganisms within a biofilm and their high mutation rate, significantly enhance the resistance of biofilms to conventional antimicrobial treatments. Peptoids are peptide‐mimics that share many features of host defence antimicrobial peptides but have increased resistance to proteases and therefore have better stability in vivo. The activity of a library of peptoids was tested against monospecies and polymicrobial bacterial/fungal biofilms. Selected peptoids showed significant bactericidal and fungicidal activity against the polymicrobial biofilms. This coupled with low cytotoxicity suggests that peptoids could offer a new option for the treatment of clinically relevant polymicrobial infections.

## Introduction

It is well recognised that clinically relevant microorganisms exist naturally as complex biofilm communities that differ substantially from their planktonic counterparts.^1^, ^2^ Biofilm organisms have a propensity for metabolic inactivity, and therefore antimicrobial agents showing promise in planktonic culture tend to be less efficacious against biofilms.^1^ The biofilm mode of growth has therefore been proposed as a mechanism for the resistance of many chronic infections to antimicrobial agents.^3^

The majority of in vitro treatment studies on biofilms focus on single‐species biofilms, whereas it is recognised that biofilms in vivo are naturally polymicrobial, and can include members of both bacterial and fungal genera. In polymicrobial infections, microorganisms have been proposed to influence each other either by exchange of molecules (sensing and signalling) or by physical contact (biofilm architecture), and this might facilitate competitive, synergistic or neutral relationships.^4^, ^5^ In addition to a growing interest in multispecies bacterial biofilms,^6^ there is also an emerging interest in the study of polymicrobial fungal–bacterial biofilms. Cross‐kingdom biofilms containing *Candida albicans* 7 have been shown to be associated with clinical infections of both biotic and abiotic surfaces (e.g. cornea^8^ and endotracheal tubes,^9^ respectively).^10^ Although the role of *C. albicans* in polymicrobial biofilm formation is likely to be complex, recent work suggests that *C. albicans* dramatically modifies the physical environment and 3 D architecture of polymicrobial biofilms^11^ as well as influencing interspecies protein expression^12^ and extracellular DNA (eDNA) release.^13^

Of particular concern in the current climate of antimicrobial resistance are the findings, from drug susceptibility studies, that fungal cells can modulate the action of antibiotics, and that bacteria can influence antifungal activity.^14^ Given the importance of polymicrobial biofilms in vivo^10^ and the increased bacterial resistance to antibiotics observed in polymicrobial biofilms containing *C. albicans*,^15^ new treatments for cross‐kingdom biofilms are urgently required. The efficacy of the innate immune system's host defence peptides (HDPs) in providing the first line of defence against infection, and their broad‐spectrum action against both bacteria and fungi has prompted us and others to investigate HDPs as templates for the design of innovative therapeutics.^16^, ^17^, ^18^, ^19^ There is particular need to identify new strategies for managing infection in order to spare conventional systemic antimicrobial drugs. Despite promising in vitro antimicrobial activities against a range of bacterial and fungal pathogens, HDPs have been shown to be susceptible to degradation by proteinases at wound and inflammatory sites,^20^, ^21^ thus potentially limiting their application as anti‐infectives. Despite the somewhat limited clinical success of such peptides,^22^ continuing advances in the design of peptide mimics and the unmet clinical need for novel antimicrobials have reinvigorated this research field.

**Figure 1:**
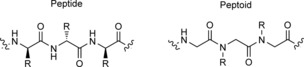
Representative structures of α‐peptide and α‐peptoid.

Oligo N‐substituted glycines (peptoids) are peptide analogues that have many of the features of HDPs, with the advantage that they are resistant to proteinases^23^ and therefore offer a new avenue for antimicrobial therapeutics. Peptoids are structural isomers of peptides, where the side‐chain functionality is attached to the nitrogen atom of the amide backbone rather than the peptide α‐carbon (Figure 1). Peptoids have been shown to be efficacious against planktonic microorganisms^24^, ^25^ and bacterial biofilms,^26^ but their activities against fungal biofilms and, in particular, polymicrobial bacterial–fungal biofilms remain to be determined.

In this study we initially screened the efficacy of a linear peptoid library against *C. albicans, Staphylococcus aureus* and *Escherichia coli* monospecies biofilms, and then determined selected peptoid efficacy against polymicrobial bacterial–fungal biofilms (*C. albicans–S. aureus* and *C. albicans–E. coli*). We used a crystal violet biofilm assay for the initial screening, and adapted a novel quantitative PCR (qPCR) approach with propidium monoazide (PMA)^27^ for determining viable organism numbers within the polymicrobial biofilms. The PMA‐qPCR assay is highly advantageous compared to traditional colony forming unit (CFU) assays, in that it allows quantification of viable but non‐culturable (VBNC) microorganisms. The PMA‐qPCR assay is therefore particularly relevant for quantification of microorganism within biofilms, which by their very nature are likely to contain VBNC microorganisms. Additionally, the crystal violet assay cannot distinguish between different species within a polymicrobial biofilm, whereas qPCR can determine viable cell counts for individual specific pathogens within polymicrobial biofilms, and therefore it offers a significant advantage in studies of this kind. To the best of our knowledge, this is also the first time that peptoids have been proven to be efficacious against fungal biofilms.

## Results and Discussion

### Peptoid design and synthesis

A library of 18 linear peptoids was synthesized to assess anti‐biofilm activity. The sequences were designed around an *NxNyNy* subunit (Table 1), repeated two, three or four times (six‐, nine‐ and twelve‐residue peptoids respectively), where *Nx* is a positively charged lysine‐type amine with various side‐chain lengths (*N*ah *N*‐(6‐aminohexyl)glycine, *N*Lys *N*‐(4‐aminobutyl)glycine and *N*ae *N*‐(2‐aminoethyl)glycine), and *Ny* is either the chiral aromatic building block *N*spe *N*‐(*S*‐phenylethyl)glycine or the achiral *N*phe *N*‐benzylglycine. Peptoids were synthesised with a repeat unit of three residues in order to induce an amphipathic structure; the bulky *N*phe and *N*spe monomers were included as these have been reported to encourage a helical structure that can lead to an improved antimicrobial activity.^28^, ^29^, ^30^, ^31^ The 18 peptoids are classified into three families (peptoids **1**–**6**, **7**–**12** and **13**–**18**) based on their positively charged building blocks (*N*ah, *N*Lys or *N*ae). All peptoids were synthesised manually on resin by the sub‐monomer method^32^ on a shaker platform at room temperature (15 min acylation steps and 15 min displacement steps). The library was purified by RP‐HPLC to greater than 95 % purity (see the Supporting Information for synthesis and characterisation).

**Table 1:** Peptoid library divided into three families (**1**–**6**; **7**–**12** and **13**–**18**) on the basis of their building blocks.

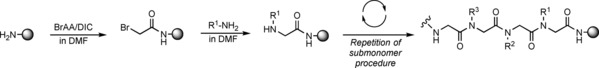				
Peptoid	Sequence	N_x_	N_y_	
**1**	(*N*Lys*N*phe*N*phe)_4_	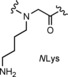	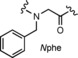	
**2**	(*N*Lys*N*phe*N*phe)_3_			
**3**	(*N*Lys*N*phe*N*phe)_2_			
**4**	(*N*Lys*N*spe*N*spe)_4_		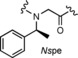	
**5**	(*N*Lys*N*spe*N*spe)_3_			
**6**	(*N*Lys*N*spe*N*spe)_2_			
				
**7**	(*N*ae*N*phe*N*phe)_4_	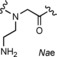	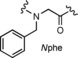	
**8**	(*N*ae*N*phe*N*phe)_3_			
**9**	(*N*ae*N*phe*N*phe)_2_			
**10**	(*N*ae*N*spe*N*spe)_4_		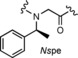	
**11**	(*N*ae*N*spe*N*spe)_3_			
**12**	(*N*ae*N*spe*N*spe)_2_			
				
**13**	(*N*ah*N*phe*N*phe)_4_	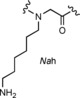	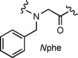	
**14**	(*N*ah*N*phe*N*phe)_3_			
**15**	(*N*ah*N*phe*N*phe)_2_			
**16**	(*N*ah*N*spe*N*spe)_4_		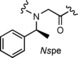	
**17**	(*N*ah*N*spe*N*spe)_3_			
**18**	(*N*ah*N*spe*N*spe)_2_			

### Peptoid efficacy against single species biofilms

**Figure 2:**
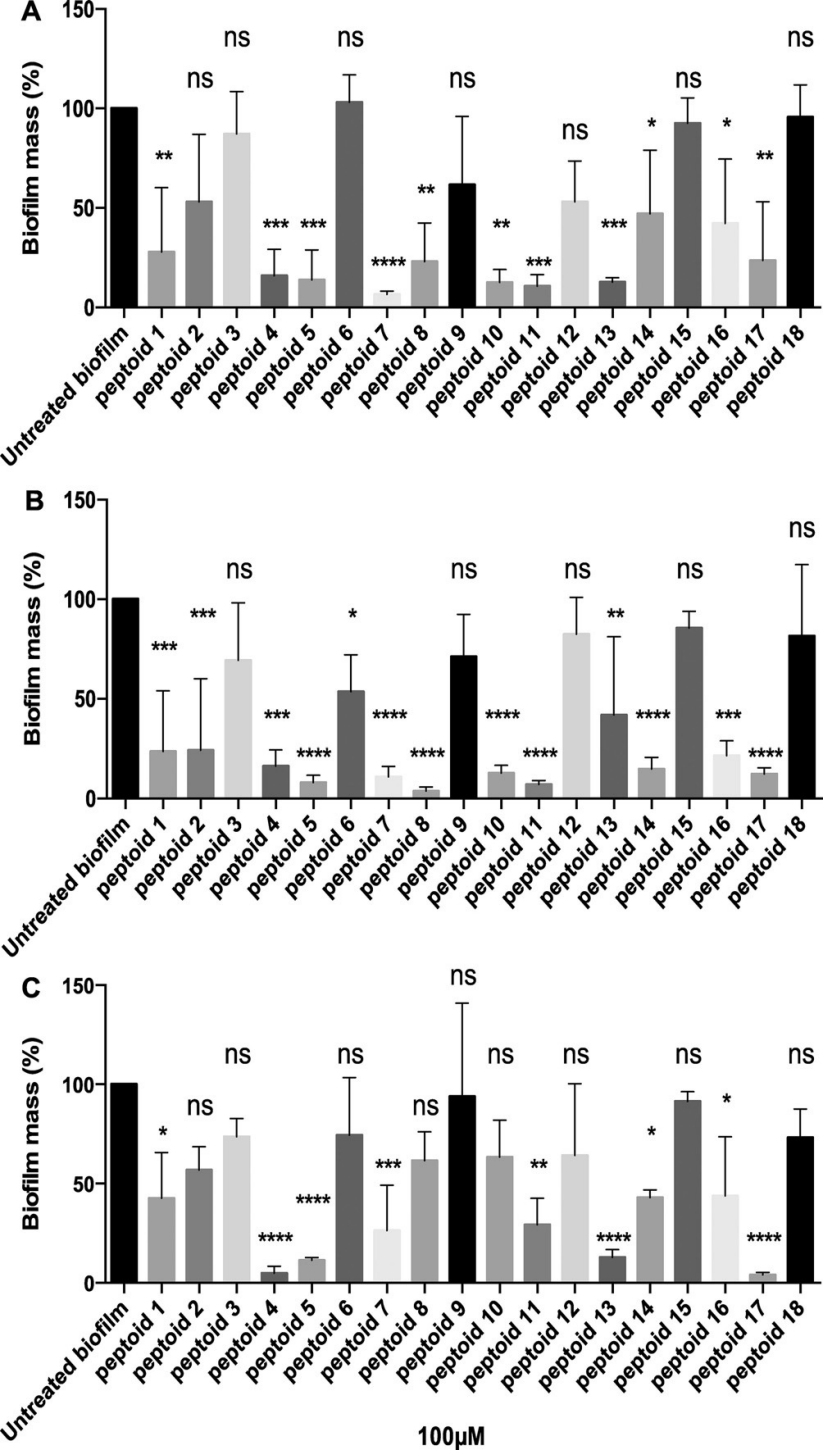
Efficacy of a family of 18 peptoids against A) *C. albicans*, B) *S. aureus*, and C) *E. coli* monospecies biofilms determined by a crystal violet assay. Biofilms were treated with peptoid (100 μm) or untreated. The results (mean±SD, *n*=3) are plotted as percent biomass relative to untreated cells. Statistical analysis was determined by one‐way ANOVA followed by Tukey's post hoc correction for multiple comparisons (ns: *p*>0.05, * *p*<0.05, ** *p*< 0.01, *** *p*<0.001, **** *p*<0.0001).

For the initial screening, the library was tested against single‐species biofilms of *C. albicans*, *S. aureus* and *E. coli*, by using a crystal violet assay (Figure 2). The 18 peptoids had differing antifungal and antibacterial activities against these single‐species biofilms, with **5**, **7**, and **17** amongst the most efficacious from each of the three peptoid families for all three single‐species biofilms.

The peptoid library contained peptoids of three different lengths: six‐, nine‐ and twelve‐residue peptoid analogues. In agreement with previous studies,^24^, ^30^, ^31^ the longest (twelve‐residue) peptoids tended to be most efficacious. They were more active against *C. albicans* than nine‐residue peptoids, and the six‐residue sequences were mostly inactive (compare **7** (dodecamer), **8** (nonamer) and **9** (hexamer)). For *S. aureus*, the longer dodecamer sequences also had good activity, but interestingly in most cases the nine‐residue analogues caused a greater reduction in biofilm mass; as found for *C. albicans*, the six monomer peptoids were inactive. For *E. coli*, the longest peptoids were the most active, except for those containing the *N*ah monomer, where the nine‐residue peptoids were most active (i.e., **13** and **17**). Direct comparisons of sequence length effects with previous reports^28^, ^29^ are complicated by the differing side‐chains used and the resulting differences in charge/length ratio.

The success of many linear antimicrobial peptides (AMPs) is attributed to their overall positive charge, which helps them to target prokaryotic cell membranes over mammalian cells,^16^ Therefore, the effect of different cationic monomers was investigated across the peptoid library. For sequences of the same length (i.e., 12 residues), peptoids containing the shortest monomer (*N*ae) tended to be more active than the longer monomer (e.g., *N*Lys, compare **1** and **7**) for all species. On the whole, peptoids containing *N*ah were less active than those containing either *N*ae or *N*Lys,^30^ although **17** was an exception. The reduced activity of the *N*ah*N*y*N*y peptoids might stem from the greater flexibility of the longer aminohexane chain.

The peptoid library was designed to include sequences containing either the chiral *N*spe or the achiral *N*phe monomer, in order to determine the effect of sequence chirality on anti‐biofilm activity. A range of chiral and achiral peptoid analogues were tested. Overall, sequences containing *N*spe were more efficacious than their achiral analogues (compare **1** and **4**) across all three biofilm species. However, for peptoids containing the shortest *N*ae monomer, there was little difference in activity between chiral and achiral peptoids (**10**–**12** and **7**–**9**, respectively) against *S. aureus* or *C. albicans*. The differences in activity for chiral and achiral members of the entire peptoid library were even less pronounced against *E. coli*.

The entire peptoid library was also screened against two representative mammalian cell lines, HaCaT and HepG2, to determine if the sequences were selective for the bacteria and fungi tested (Supporting Information). The majority of the peptoids showed ED_50_ values above100 μm against both, this indicating minimal cytotoxicity on model human keratinocyte and endothelial cells. The only sequences that showed toxicity were the 12‐residue peptoids containing the chiral *N*spe monomer (**4**, **10** and **16**).

Although it is generally acknowledged that peptoids are inherently resistant to proteolysis,^23^, ^33^ we compared the tryptic digestion profile of **7** against the naturally occurring alpha helical peptide LL‐37. Peptoid **7** showed no degradation following treatment with trypsin for 24 h, whereas LL‐37 was degraded to peptide fragments (Supporting Information).

### Peptoid efficacy against polymicrobial biofilms

Although widely used in biofilm assays, the crystal violet assay detects both live and dead organisms, in addition to matrix components. Conventional CFU assays have been reported to underestimate live cell numbers because they cannot quantify VBNC cells.^34^, ^35^ Furthermore, microorganisms have been shown to enter the VBNC sate when exposed to antibiotic treatment.^36^ Therefore the use of non‐cultivation‐based assays to evaluate the efficacy of novel antimicrobials is particularly appealing for studying microorganisms in biofilm form. In this study, we developed a novel PCR method to selectively and quantitatively determine fungicidal and bactericidal activity against both monospecies and polymicrobial biofilms.

PMA is a photo‐reactive dye with a high affinity for DNA, with which it forms a covalent linkage upon exposure to intense visible light. The use of PMA allows qPCR quantification of DNA from living cells only, because PMA binds covalently to DNA that lacks the protection of a cell membrane in viable microorganism^27^ and prevents DNA amplification by qPCR. As PMA discriminates between live and dead cells on the basis of membrane integrity,^37^ its addition to the qPCR protocol is particularly suitable for quantifying the efficacy of membrane‐targeting agents such as peptoids.

**Figure 3:**
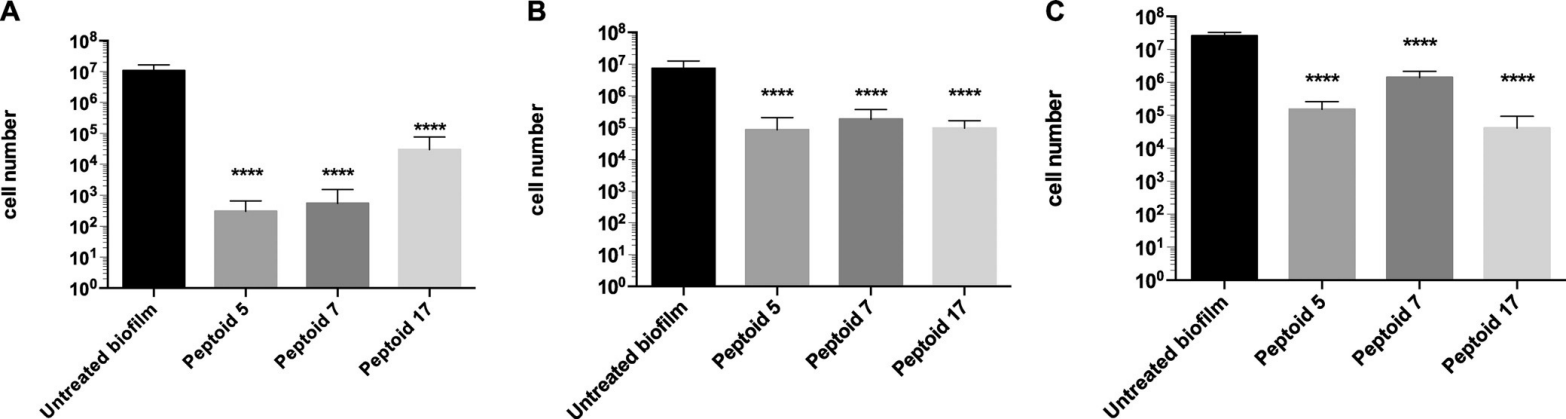
PMA‐qPCR quantification of cell number following peptoid treatment of monospecies biofilms of A) *C. albicans*, B) *S. aureus* and C) *E. coli* treated with 100 μm **5**, **7** and **17** (**** *p*<0.0001; one‐way ANOVA followed by Tukey's post hoc correction for multiple comparisons, mean±SD, *n*=3).

Three peptoids (**5** (*N*Lys*N*spe*N*spe)_3_, **7** (*N*ae*N*phe*N*phe)_4_, and **17** (*N*ah*N*spe*N*spe)_3_) were chosen, as they were some of the most active peptoids in the single‐species crystal violet assays and showed negligible toxicity to the two mammalian cell lines. All monospecies biofilms treated with these peptoids showed significant reduction in cell number by the novel PMA‐qPCR method, thus indicating strong bactericidal and fungicidal activity (Figure 3). The three peptoids had a similar efficacy against *S. aureus*: cell numbers reduced by approximately two orders of magnitude, with **5** and **7** showing even better effects (reduction by over four orders of magnitude against this microorganism). In agreement with the crystal violet assay results, the *N*ah‐containing peptoid **17** did not perform quite as well against *C. albicans*, although it still caused a significant reduction in the fungal biofilm. Conversely, in the *E. coli* monospecies biofilms, **17** was the most effective.

**Figure 4:**
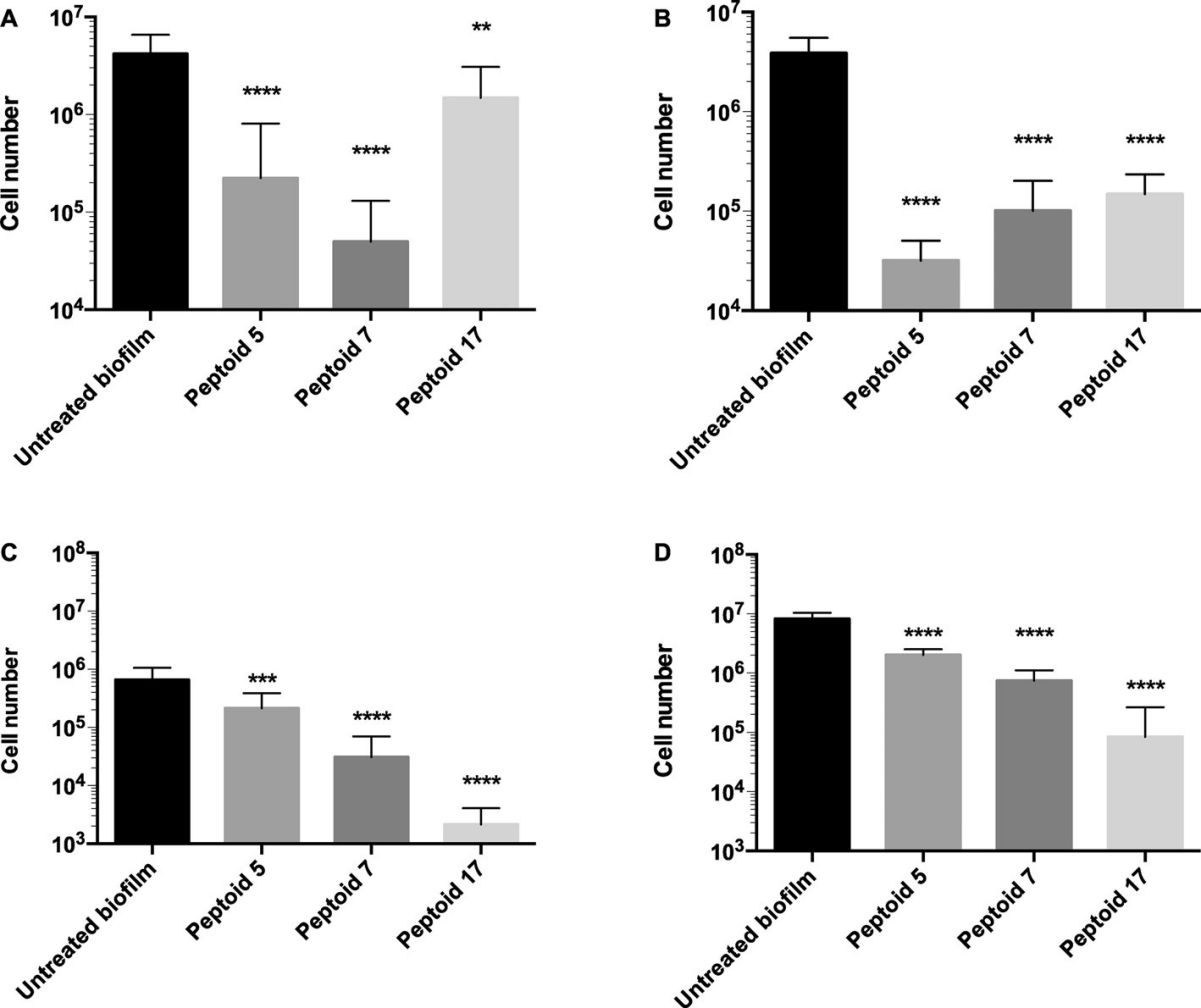
PMA‐qPCR quantification of cell number following treatment by 100 μm **5**, **7** and **17** of polymicrobial biofilms of *C. albicans* with either *S. aureus* or *E. coli*: A) *C. albicans* and B) *S. aureus* within the same biofilm, and C) *C. albicans* and D) *E. coli* within the same biofilm (one‐way ANOVA followed by Tukey's post hoc correction for multiple comparisons, ** *p*<0.01, **** *p*<0.0001; mean±SD, *n*=3).

Peptoids **5**, **7** and **17** were also tested at 100 μm against mixed‐species biofilms of *C. albicans* and either *S. aureus* or *E. coli*. (Figure 4). In the latter, the *C. albicans* cell number reduced more than for *E. coli*. The *N*ah‐containing peptoid (**17**) was the most active. In the former, cell numbers were reduced more for bacteria than fungi. Peptoid **5** was better able to reduce the cell count of *S. aureus*, and **7** showed the greatest reduction in *C. albicans. C. albicans* appeared to be less susceptible to **17** when in a biofilm with *S. aureus*, but this did not appear to be the case with *E. coli*. As there has been very limited work on peptoid efficacy in polymicrobial biofilms, it remains to be determined if this is a unique phenomenon.

In order to look in more detail at the activities of **5**, **7** and **17**, cross‐kingdom biofilms were treated at 10–100 μm and assessed by PMA‐qPCR (Supporting Information). In the mixed‐species biofilms, all three peptoids significantly reduced the cell numbers of *S. aureus* by almost 100 %, even at 10 μm. Reduction in cell viability for *E. coli* or *C. albicans* was less efficacious at 10 μm, but considerable reductions were seen at 25 and 50 μm. For all three species in the cross‐kingdom biofilms, **17** showed the greatest reduction in cell numbers.

### Effect of peptoids on microbial cell membranes

The majority of AMPs exert their antimicrobial effects by disruption of cellular membranes. It is thought that as linear antimicrobial peptoids (such as those studied here) are structurally very similar to this class of AMP, they might also exert their biological mode of action by cell membrane disruption. However, only a few studies have provided experimental evidence to support this hypothesis.^38^, ^39^, ^40^, ^41^, ^42^

**Figure 5:**
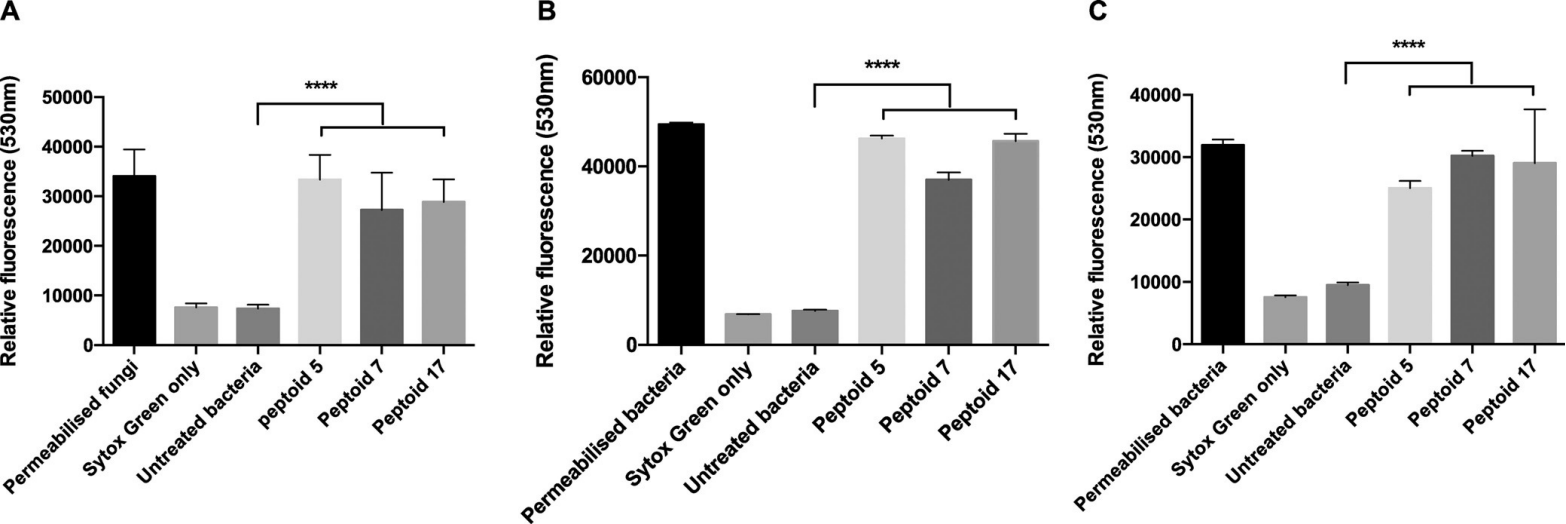
Propensity for **5**, **7** and **17** to permeabilise the microbial membranes of A) *C. albicans*, B) *E. coli* and C) *S. aureus*, as determined by a SYTOX Green assay. The data were compared with untreated cells, and the results (mean±SD, *n*=3) were analysed by ANOVA followed by Tukey's post hoc correction for multiple comparisons (**** *p*<0.0001). Cells permeabilised by heat treatment were used as positive controls.

In order to help elucidate the mode of action of the peptoids studied in the qPCR experiments and to verify the use of the PMA‐qPCR assay (depends on the integrity of the cell membrane), membrane permeabilisation assays were performed with 100 μm **5**, **7** and **17** against *C. albicans*, *S. aureus* and *E. coli*. These assays used the dye SYTOX Green, which is able to bind nucleic acids but is impermeable to living eukaryotic and prokaryotic cells. Therefore, this dye is routinely used to assess the integrity of cell membranes. If a microbial cell membrane has been compromised, for example by treatment with a peptoid, SYTOX Green can bind to the cellular nucleic acids. This association increases the fluorescence of the dye and renders cells with compromised membranes as brightly green fluorescent.^43^, ^44^ The propensities of **5**, **7** and **17** to permeabilise cell membranes was assessed (Figure 5). The data clearly show large increases in fluorescence, thus demonstrating that all three peptoids caused cell‐membrane permeabilisation in *C. albicans*, *E. coli* and *S. aureus*.

The peptoids with the greatest antimicrobial effect against single‐species biofilms (Figure 3) also caused the greatest membrane permeabilisation in the SYTOX Green assay (Figure 5). For example, against *C. albicans*, **5** caused the greatest reduction in cell number and the greatest increase in fluorescence. The results strongly support that the microbial action of **5**, **7** and **17** is membrane disruption, but additional action by a secondary intracellular target cannot be ruled out.

## Conclusion

Peptoids, an emerging class of peptidomimetics that have previously been tested principally for their activity against bacteria in planktonic and biofilm form,^26^, ^28^, ^29^, ^45^, ^46^ were shown for the first time to be efficacious against *C. albicans* biofilms and against cross‐kingdom polymicrobial biofilms. We have shown the in vitro effectiveness of a peptoid library against both single species *C. albicans, S. aureus* and *E. coli* biofilms and we also report their excellent fungicidal and bactericidal activity in polymicrobial biofilms by using PMA‐modified qPCR. SYTOX Green membrane‐leakage assays identified that the cell membranes of *C. albicans*, *S. aureus* and *E. coli* are the likely targets through which the peptoids screened exert their antimicrobial mode of action.

The peptoid library showed similar activity patterns across the three species tested by crystal violet assay, that is, peptoids that were active at some level against one species also tended to be active against the others. However, these peptoids generally caused the greatest reduction in biofilm mass against *S. aureus*, followed by *C. albicans,* with *E. coli* biofilms showing the least reduction in biofilm mass following peptoid treatment. Planktonic *E. coli* has also proven more difficult to treat than Gram‐positive bacteria in previous studies on peptoid efficacy.^24^, ^25^

From the qPCR data, **17** (*N*ah*N*spe*N*spe)_3_ was identified as the most promising candidate. This peptoid has low toxicity, significant activity against *S. aureus* in a mixed‐species biofilm with *C. albicans* at 10 μm, and it also shows good activity against both species in the *C. albicans* and *E. coli* biofilm at higher concentrations.

Finally, peptoids potentially have an advantage over conventional antifungals and antibacterials in that their proposed biological mode of action (disruption of cellular membranes) limits the emergence of resistance as this would require the fungi and bacteria to undertake the arduous task of altering cellular membrane composition. Furthermore, their enhanced stability towards proteolytic degradation means that peptoids, rather than peptides, might represent a more realistic class of molecule for the development of new therapeutics.

## Experimental Section

**Peptoid synthesis**: Details can be found in the Supporting Information. Fmoc‐protected Rink amide resin (typically 100 mg, 0.1 mmol; 0.6–0.8 mmol g^−1^ loading) was swollen in DMF (at least 1 h at room temperature, overnight preferred) in a 20 mL polypropylene syringe fitted with two polyethylene frits (Crawford Scientific). The resin was deprotected with piperidine (20 % *v/v* in DMF, 2×20 min) and washed with DMF (3×2 mL). The resin was treated with bromoacetic acid (8 equiv with respect to the resin, 2 m in DMF) and DIC (8 equiv, 2 m in DMF) for 15 min at 50 °C on a heated shaker at 400 rpm. The resin was washed with DMF (3×2 mL), before the desired amine sub‐monomer was added (4 equiv, 1 m in DMF) and allowed to react for 15 min at 50 °C on the shaker. The resin was again washed with DMF (3×2 mL), and the bromoacetylation and amine displacement steps were repeated until the final sub‐monomer had been added (i.e. desired peptoid sequence obtained). The resin was shrunk in diethyl ether (3 mL), and final cleavage from resin was achieved by using a TFA cleavage cocktail (4 mL, TFA/TIPS/H_2_O, 95:2.5:2.5) on the shaker (400 rpm) for 60 min. The resin was removed by filtration, and the cleavage cocktail was removed in vacuo. The crude product was precipitated in diethyl ether (30 mL), and the precipitate was retrieved by centrifugation (447 *g*, 15 min). The ether phase was decanted, and the crude product was dissolved in a mixture of acidified H_2_O and MeCN and lyophilised before purification by RP‐HPLC and subsequent characterisation (see the Supporting Information for equipment, procedures and data).

**Peptoid cytotoxicity studies**: All 18 peptoids were tested for cytotoxicity against HepG2 epithelial (Sigma–Aldrich) and HaCaT keratinocyte (ThermoFisher) cell lines, Cytotoxicity analyses were performed in Costar 96‐well plates (Thermo Fisher Scientific) with alamarBlue (Invitrogen/Thermo Fisher Scientific) for cell viability detection as described by the manufacturer. HepG2 and HaCaT cells were grown at 37 °C under CO_2_ (5 %) in DMEM (high glucose) supplemented with heat‐inactivated foetal bovine sera (FBS, 10 %; Biosera, Uckfield, UK) and penicillin/streptomycin (1 %). Cells were counted in a Neubauer Improved Haemocytometer. HepG2 or HaCaT cells were seeded 24 h prior to treatment in 96‐well plates (2×10^4^ cells per well in 100 μL). After 24 h, cells were incubated in triplicate with a dilution series of compound (2–100 μm from 5 mm stock solutions in DMSO) in the medium (50 μL) for 1 h. An aliquot (40 μL) was removed from each well, before the addition of medium (90 μL) followed by incubation for 24 h (37 °C, 5 % CO_2_). Then, alamarBlue (10 μL) was added to each well before incubation (2 h for Hep G2, 1 h for HaCaT) prior to assessing cell viability in a fluorescent plate reader (Biotek, Winooski, VT; *λ*_ex_=560 nm, *λ*_em_=600 nm). All data were measured in triplicate on a minimum of two occasions.

**Proteolytic stability studies**: Peptoid stability was confirmed by comparing the tryptic digestion of **7** with LL‐37 (Innovagen AB, Lund, Sweden). Stock solutions (5 mg mL^−1^) of **7** and LL‐37 were prepared, and samples (7.5 μL) were incubated with trypsin (0.5 μg mL^−1^) and incubation buffer (37.5 μL, Tris**⋅**HCl (50 mm, pH 7.8) containing CaCl_2_ (9 mm)). After 0 h (for mass spectrometric verification) or 24 h, the reaction mixture was acidified by addition of TFA (10 %, 50 μL) to denature the enzyme and stop enzyme activity. The samples were air‐dried and then reconstituted in acetonitrile/water/TFA (50 μL, 40:59.5:0.5, *v/v/v*), and an aliquot (1 μL) was placed onto a stainless steel MALDI target. Samples were covered immediately with matrix (1 μL, α‐cyano‐4‐hydroxycinnamic acid (53 mm in acetonitrile/water/TFA, 70:29.98:0.02, *v/v/v*)). MALDI‐TOF MS was performed on a linear TOF Voyager DE‐mass spectrometer (PerSeptive Biosystems, Farmingham, MA, USA). The samples were analysed in positive detection mode, and internal mass calibration with known standards established the mass accuracy to ±0.1 %. Fifty laser scans were averaged for each sample, and variable laser intensity was used to ensure the most representative mass spectra for the wells.

**Microorganism strains and growth conditions**: *C. albicans* (NCTC 3179) was subcultured aerobically on Sabouraud agar plates, prepared by using Sabouraud dextrose powder and agar (Oxoid, ThermoFisher) and propagated in yeast peptone dextrose broth. *E. coli* (ATCC 29522) and *S. aureus* (NCTC 6571) were grown on blood agar plates (Fanin, Dublin, Ireland) and propagated in brain heart infusion (BHI) broth (Oxoid, ThermoFisher).

**Preparation and treatment of single‐species biofilms**: Overnight cultures of *C. albicans* were washed and resuspended (1.0×10^6^ cells per mL) in RPMI‐1640 (Sigma–Aldrich). Overnight cultures of *S. aureus* or *E. coli* were washed and resuspended (5.0×10^6^ cells per mL) in BHI broth. Samples (100 μL) were added to microtitre plate wells (Thermo Fisher Scientific), and a biofilm was allowed to form for 4 h. Wells were washed with PBS (3×200 μL) to facilitate removal of planktonic cells, and the biofilms were then treated with **1**–**18** (100 μm in the appropriate medium). Plates were incubated for a further 24 h to allow biofilm maturation. After removal of planktonic cells by washing, biofilms were quantified by the crystal violet assay or by PMA‐qPCR.

**Preparation and treatment of polymicrobial biofilms**: Overnight cultures of *C. albicans* were prepared in microtitre plates as above, and allowed to adhere for 4 h to facilitate initial biofilm formation. Planktonic *C. albicans* cells were removed as above before the addition of *S. aureus* or *E. coli* (100 μL, 5.0×10^6^ cells per mL). Bacteria were allowed to adhere to the *C. albicans* biofilms for 4 h to facilitate polymicrobial biofilm formation. Following a washing step, the biofilms were then treated with peptoid (10–100 μm), and incubated for a further 24 h to allow biofilm maturation. Wells were washed as above, and the polymicrobial biofilms were quantified by PMA‐modified qPCR.

**Biofilm quantification by crystal violet assay**: Washed biofilms were fixed with methanol (100 μL) for 10 min. Following removal of methanol, the wells were air dried and stained with crystal violet solution (Clin‐Tech, Guildford, UK) for 20 min at room temperature. Excess stain was removed by washing, then the plate was air‐dried and bound crystal violet was re‐solubilised in acetic acid (33 %, 160 μL) prior to reading at 570 nm in a GENios microtitre plate reader (Tecan, Zürich, Switzerland).

**Biofilm quantification by PMA‐modified qPCR**: In order to determine the bactericidal and fungicidal activity of peptoids against single‐species and polymicrobial biofilms, the biofilms were detached from the microtitre plate wells prior to quantification. The wells were washed as above, then BHI broth (100 μL) was added and the plate was sealed. Biofilm detachment was achieved by sonication for 5 min in an ultrasonic bath (Dawe, Hayes, Middlesex, UK). The remaining cells were then collected in BHI (80 μL). PMA (20 μL, 2 mm in broth;^47^ Biotium, Fremont, CA) was added to the biofilm suspensions (180 μL) and incubated (37 °C, 5 min) prior to photoactivation with a broad‐spectrum LED flood light (60 LED, 0.1 W) placed 15 cm from the tubes (mixed by inversion during the 20 min photoactivation step).^48^ DNA was extracted by using a microLYSIS‐Plus kit (Microzone, Haywards Heath, UK), and qPCR was performed in a Mx3005P qPCR System (Agilent Technologies).^49^, ^50^, ^51^ See the Tables in the Supporting Information.

**Generation of standard curves for PMA‐qPCR**: For quantification of *C. albicans, S. aureus* and *E. coli* in single‐species and polymicrobial biofilms, DNA standards were prepared by extraction of DNA from planktonic organisms with the microLYSIS‐Plus kit and purification with a DNeasy kit (Qiagen). DNA standards (10^1^–10^6^ cells) were used in PMA‐qPCR assays to generate standard curves from which the numbers of living organisms within the biofilms could be determined.

**SYTOX Green assay**: Briefly, mid‐log‐phase microorganism cultures were adjusted to the appropriate concentration (OD_600_=0.7 for *S. aureus* and *E. coli*, OD_600_=2.0 for *C. albicans*). A microorganism suspension in Mueller Hinton broth (MHB,50 μL) was added to each well of a 96‐well black flat‐bottomed plate. Peptoids (50 μL, final 100 μm) were added to the wells then SYTOX Green (ThermoFisher; final 5 μm) was added. The plate was covered, protected from light and incubated for 2 h at 37 °C. Heat‐treated microorganisms (99 °C for 10 min to permeabilise membranes) served as positive controls. Bacteria without peptoid were negative controls. Wells containing only SYTOX Green were included to quantify background fluorescence. Wells containing SYTOX Green and peptoid were included to confirm no interaction between the SYTOX Green and peptoid leading to non‐specific fluorescence. The plate was read on a SpectraMax Gemini X fluorimeter (Molecular Devices, Sunnyvale, CA; *λ*_ex_=480 nm, *λ*_em_=530 nm).

**Statistical analysis**: The susceptibility of *C. albicans*, *S. aureus* and *E. coli* in both single‐species and polymicrobial biofilms to novel peptoids was determined by biofilm inhibition assays. All data from three independent experiments were subject to statistical analysis by one‐way ANOVA followed by Tukey's post hoc correction for multiple comparisons.

## Supporting information

As a service to our authors and readers, this journal provides supporting information supplied by the authors. Such materials are peer reviewed and may be re‐organized for online delivery, but are not copy‐edited or typeset. Technical support issues arising from supporting information (other than missing files) should be addressed to the authors.

Supplementary
