# Supplementary material for: Peptoid Efficacy against Polymicrobial Biofilms Determined by Using Propidium Monoazide‐Modified Quantitative PCR
Source: Chembiochem. 2016 Nov 30;18(1):111–8. doi: 10.1002/cbic.201600381 (PMC6680226; doi:10.1002/cbic.201600381)
Supplement: Supplementary file 1 — Supplementary [file CBIC-18-111-s001.pdf]

CHEMBIOCHEM

Supporting Information

Peptoid Efficacy against Polymicrobial Biofilms Determined by Using Propidium Monoazide-Modified Quantitative PCR

Yu Luo+,[a] Hannah L. Bolt+,[b] Gabriela A. Eggimann,[b] Danny F. McAuley,[a] Ronan McMullan,[a] Tanya Curran,[c] Mei Zhou,[d] Professor Colin A. B. Jahoda,[e] Steven L. Cobb,*[b] and Fionnuala T. Lundy*[a]

cbic_201600381_sm_miscellaneous_information.pdf

Contents

Contents 1

1. Procedure 2

 1.1. Materials and Reagents 2

 1.2. Peptoid Synthesis 2

2. Characterisation 4

 2.1. Characterisation of building blocks and peptoids used in this study 4

 2.2. Accurate Mass Data 5

 2.3. Representative Analytical RP-HPLC 6

 2.4. Chemical and Physical Data for Peptoid Library 10

 2.5. LC-MS Spectra for Peptoid Library 11

3. qPCR reaction mixes and conditions 21

4. Proteolytic Stability Study 26

5. Toxicity to mammalian cell lines 28

1. Procedure

1.1. Materials and Reagents

Abbreviations for reagents are as follows: *tert*-butoxycarbonyl (Boc); 9-fluorenylmethoxylcarbonyl (Fmoc); trifluoroacetic acid (TFA); triisopropylsilyl (TIPS); *N,N*-dimethylformamide (DMF); *N,N*-diisopropylcarbodiimide (DIC); dimethylsulphoxide (DMSO); bromoacetic acid (BrAA). Solvents and reagents were purchased from commercial sources and used without further purification unless otherwise noted. Rink amide resin (typical loading level 0.6-0.8 mmol g^{-1}) was purchased from Merck4Biosciences. DMF was purchased from AGTC Bioproducts (National Diagnostics). Piperidine, bromoacetic acid and TFA were purchased from Sigma Aldrich. The amine building blocks were sourced from Sigma Aldrich or TCI Europe.

1.2. Peptoid Synthesis

Synthesis as previously described and shown in Figure S1 (G. A. Eggimann, H. L. Bolt, P. W. Denny, S. L. Cobb, *ChemMedChem,* 2015, **10**, 214-214). Fmoc-protected Rink Amide resin (normally 100 mg, 0.1 mmol, typical loading between 0.6–0.8 mmol g^{-1}) was swollen in DMF (at least 1 hour at room temperature, overnight preferred) in a 20 mL polypropylene syringe fitted with two polyethylene frits (Crawford Scientific). The resin was deprotected with piperidine (20% in DMF v/v, 2 x 20 min) and washed with DMF (3 x 2mL). The resin was treated with bromoacetic acid (8 eq. with respect to the resin, 2M in DMF) and DIC (8 eq., 2M in DMF) for 15 minutes at 50 ˚C on a heated shaker at 400 rpm. The resin was washed with DMF (3 x 2 mL), before the desired amine sub-monomer was added (4 eq., 1M in DMF) and allowed to react for 15 minutes at 50 ˚C on the shaker. The resin was again washed with DMF (3 x 2 mL) and the bromoacetylation and amine displacement steps were repeated until the final sub-monomer had been added and the desired peptoid sequence had been obtained. The resin was shrunk in diethyl ether (3 ml) and final cleavage from resin was achieved using a TFA cleavage cocktail (4 ml, TFA:TIPS:H_2O, 95:2.5:2.5) on the shaker at 400 rpm for 60 minutes. The resin was removed by filtration and the cleavage cocktail removed *in vacuuo*. The crude product was precipitated in diethyl ether (30 mL) and the precipitate retrieved by centrifuge for 15 min at 5,000 rpm. The ether phase was decanted and the crude product dissolved in a mixture of acidified H_2O and MeCN and lyophilised before purification. Preparative RP-HPLC was performed with a semi-preparative Perkin Elmer Series 200 lc pump fitted with a 785A UV/Vis detector using a SB-Analytical ODH-S optimal column (250 × 10 mm, 5 µm); flow rate 2 ml min^{-1}; λ = 250 nm, typical linear gradient elution 0-50% of solvent B over 60 min (*A* = 0.1% TFA in 95% H_2O and 5% MeCN, *B* = 0.1% TFA in 5% H_2O and 95% MeCN). Analytical RP-HPLC was performed with a Perkin Elmer Series 200 lc pump fitted with a 785A UV/Vis detector using a SB-Analytical ODH-S optimal column (100 × 1.6 mm, 3.5 µm); flow rate 1 ml min^{-1}; λ = 220 nm, linear gradient elution 0-100% of solvent B over 30 min (*A* = 0.05% TFA, 95% H_2O, 5% MeCN, *B* = 0.03% TFA, 5% H_2O, 95% MeCN).

Peptoids were characterised by accurate LC-MS (QToF mass spectrometer and an Acquity UPLC from Waters Ltd.) using an Acquity UPLC BEH C8 1.7µm (2.1mm × 50mm) column with a flow rate of 0.6 ml min^{-1} and a linear gradient of 5-95% of solvent B over 3.8 min (*A* = 0.1%

formic acid in H_2O, *B* = 0.1% formic acid in MeCN). Peptide identities were also confirmed by MALDI-TOF mass spectra analysis (Autoflex II ToF/ToF mass spectrometer Bruker Daltonik GmBH) operating in positive ion mode using an α-cyano-4-hydroxycinnamic acid (CHCA) matrix. Data processing was done with MestReNova Version 8.1.

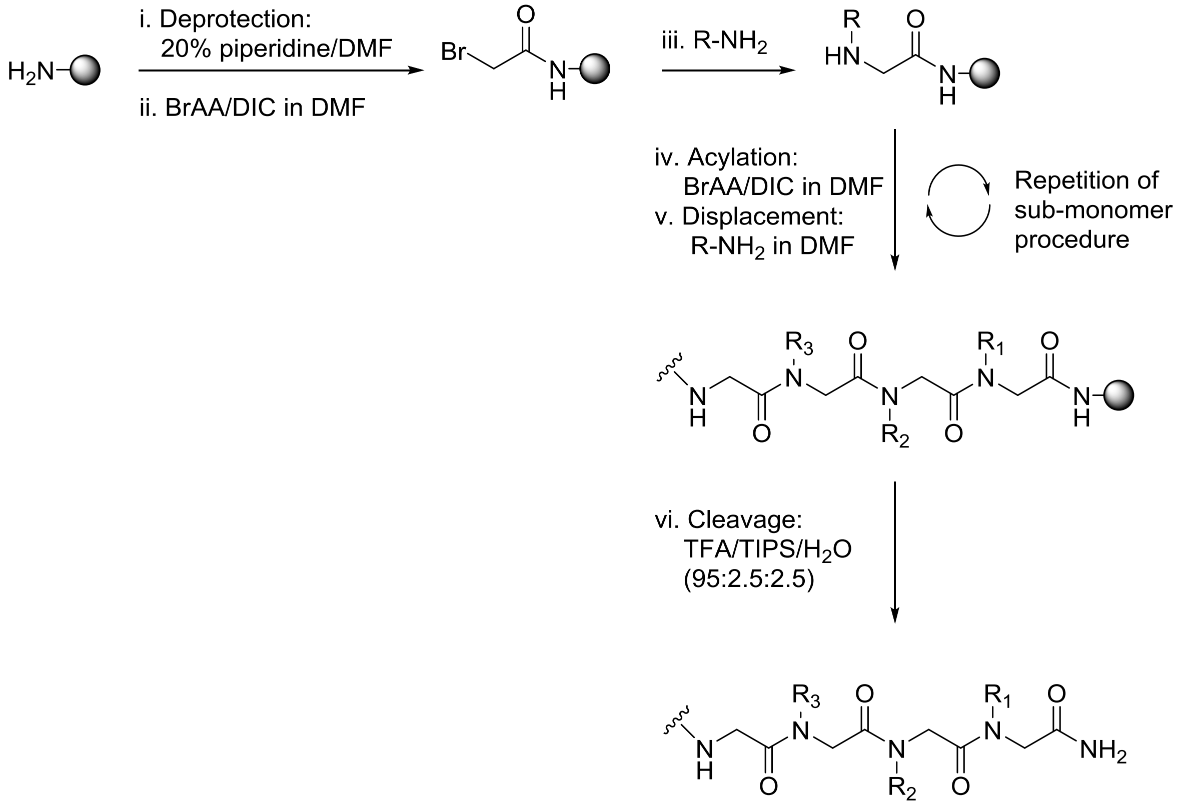

Figure S1. The submonomer method of peptoid synthesis on solid phase; [i] swelling and deprotection of resin; [ii] acylation using bromoacetic acid and DIC in DMF; [iii] displacement step (primary amine in DMF); [iv/v] successive cycles of acylation and displacement; [vi] acidic TFA cleavage of product from resin.

2. Characterisation

2.1. Characterisation of building blocks and peptoids used in this study

Table S1: The abbreviations used for the peptoid monomers synthesised in this study, and the amines that they were derived from.

Monomer	Chemical Structure	Amine Sub-monomer
***N*Lys**<br>*N*-(4-aminobutyl) glycine	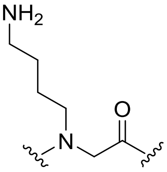	*N*-Boc-1,4-diaminobutane
***N*ah**<br>*N*-(4-aminohexyl) glycine	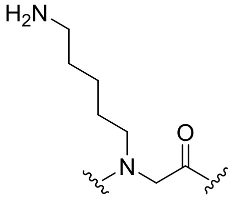	*N*-Boc-1,4-diaminohexane
***N*ae**<br>*N*-(4-aminoethyl) glycine	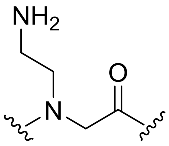	*N*-Boc-1,4-diaminoethane
***N*phe**<br>*N*-(phenylmethyl) glycine	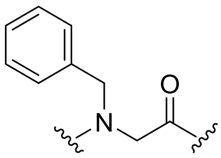	Benzylamine
***N*spe**<br>N(*S*-phenylethyl) glycine	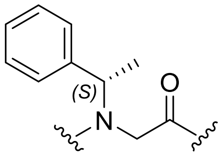	*(S)*-(−)-α-Methylbenzylamine

2.2. Accurate Mass Data

Table S2. Accurate mass spectrometry data and data from analytical RP-HPLC for representative compounds of this peptoid library. Accurate mass data calculated for the $[M+2H]^{2+}$ ion.

	Sequence	Molecular Formula	Mass Calculated $[M+2H]^{2+}$	Mass Observed $[M+2H]^{2+}$
**1**	$(Nah\textit{N}phe\textit{N}phe)_4$	$C_{104}H_{139}N_{17}O_{12}$	910.0473	910.0468
**2**	$(Nah\textit{N}phe\textit{N}phe)_3$	$C_{78}H_{105}N_{13}O_9$	684.9157	684.9141
**3**	$(Nah\textit{N}phe\textit{N}phe)_2$	$C_{52}H_{71}N_9O_6$	918.5605	918.5634
**4**	$(NLys\textit{N}phe\textit{N}phe)_4$	$C_{96}H_{123}N_{17}O_{12}$	853.9847	853.9848
**5**	$(NLys\textit{N}phe\textit{N}phe)_3$	$C_{72}H_{93}N_{13}O_9$	642.8688	642.8666
**6**	$(NLys\textit{N}phe\textit{N}phe)_2$	$C_{48}H_{63}N_9O_6$	431.7529	431.7513
**7**	$(Nae\textit{N}phe\textit{N}phe)_4$	$C_{88}H_{107}N_{17}O_{12}$	797.9221	797.9189
**8**	$(Nae\textit{N}phe\textit{N}phe)_3$	$C_{66}H_{81}N_{13}O_9$	600.8218	600.8185
**9**	$(Nae\textit{N}phe\textit{N}phe)_2$	$C_{44}H_{55}N_9O_6$	806.4354	806.437
**10**	$(Nah\textit{N}spe\textit{N}spe)_4$	$C_{112}H_{155}N_{17}O_{12}$	966.1099	966.1109
**11**	$(Nah\textit{N}spe\textit{N}spe)_3$	$C_{84}H_{117}N_{13}O_9$	726.9627	726.9601
**12**	$(Nah\textit{N}spe\textit{N}spe)_2$	$C_{56}H_{79}N_9O_6$	974.6232	974.6246
**13**	$(NLys\textit{N}spe\textit{N}spe)_4$	$C_{104}H_{139}N_{17}O_{12}$	910.0473	910.0494
**14**	$(NLys\textit{N}spe\textit{N}spe)_3$	$C_{78}H_{105}N_{13}O_9$	684.9157	684.9142
**15**	$(NLys\textit{N}spe\textit{N}spe)_2$	$C_{52}H_{71}N_9O_6$	459.7842	459.7801
**16**	$(Nae\textit{N}spe\textit{N}spe)_4$	$C_{96}H_{123}N_{17}O_{12}$	853.9847	853.9841
**17**	$(Nae\textit{N}spe\textit{N}spe)_3$	$C_{72}H_{93}N_{13}O_9$	642.8688	642.8675
**18**	$(Nae\textit{N}spe\textit{N}spe)_2$	$C_{48}H_{63}N_9O_6$	862.4980	862.4994

2.3. Representative Analytical RP-HPLC

Table S3. Data from analytical RP-HPLC for representative compounds of this peptoid library. Analytical HPLC gradient: 0 – 100% solvent B over 30 min at 220 nm (where solvent A = 95% H_2O, 5% MeCN, 0.05 % TFA; solvent B = 95% MeCN, 5% H_2O, 0.03% TFA).

	Sequence	RP-HPLC Retention *(min)*	RP-HPLC Approx. Purity (%)
**1**	(*N*ah*N*phe*N*phe)$_4$	14.7	>95
**7**	(*N*ae*N*phe*N*phe)$_4$	15.2	>95
**13**	(*N*Lys*N*spe*N*spe)$_4$	16.2	>95
**14**	(*N*Lys*N*spe*N*spe)$_3$	15.3	>95
**15**	(*N*Lys*N*spe*N*spe)$_4$	14.4	>95
**16**	(*N*ae*N*spe*N*spe)$_4$	16.0	>95

Analytical HPLC trace Peptoid 1 (*N*ah*N*phe*N*phe)$_2$

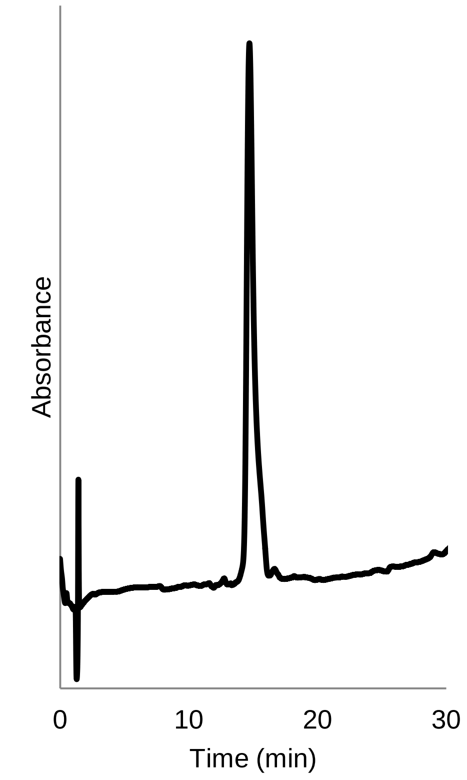

Analytical HPLC trace Peptoid 7 (*N*ae*N*phe*N*phe)$_4$

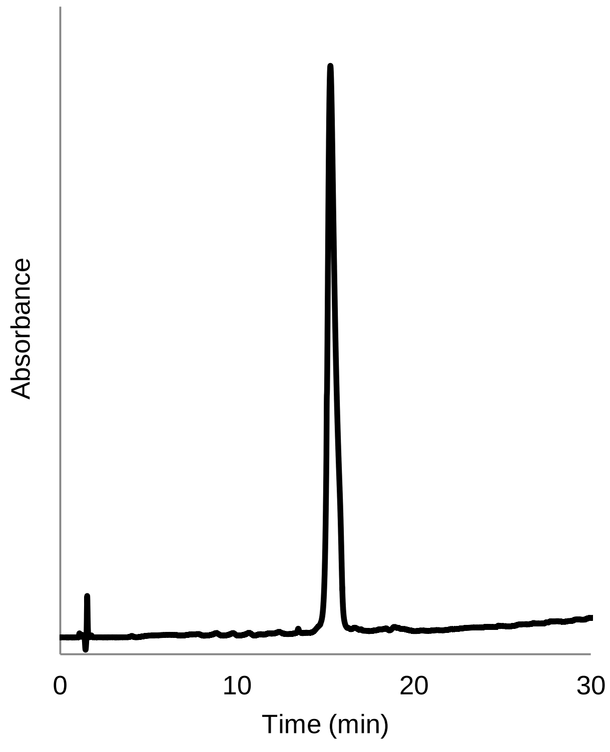

Analytical HPLC trace Peptoid 13 (*N*Lys*N*spe*N*spe)$_4$

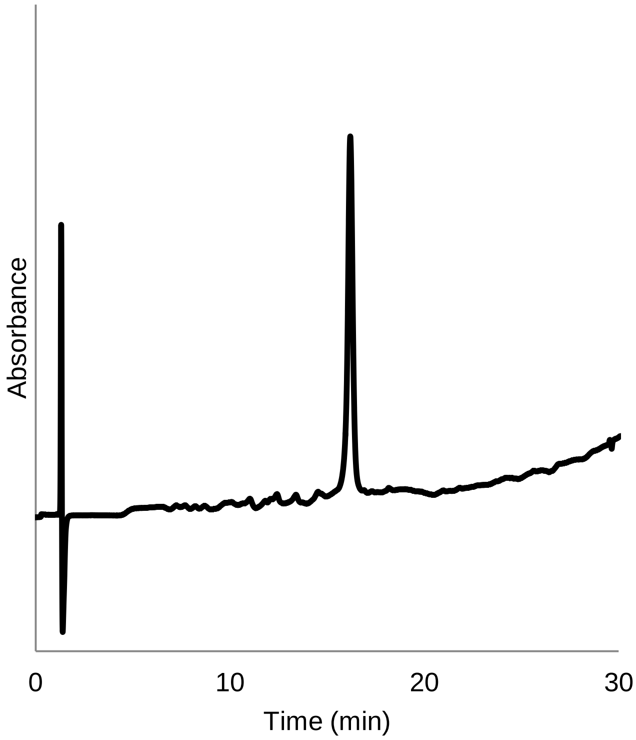

Analytical HPLC trace Peptoid 14 (*N*Lys*N*spe*N*spe)$_3$

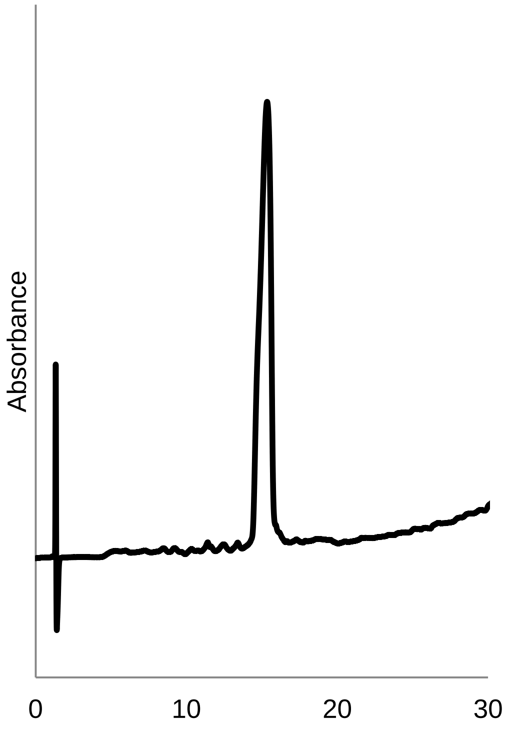

Analytical HPLC trace Peptoid 15 (*N*Lys*N*spe*N*spe)$_2$

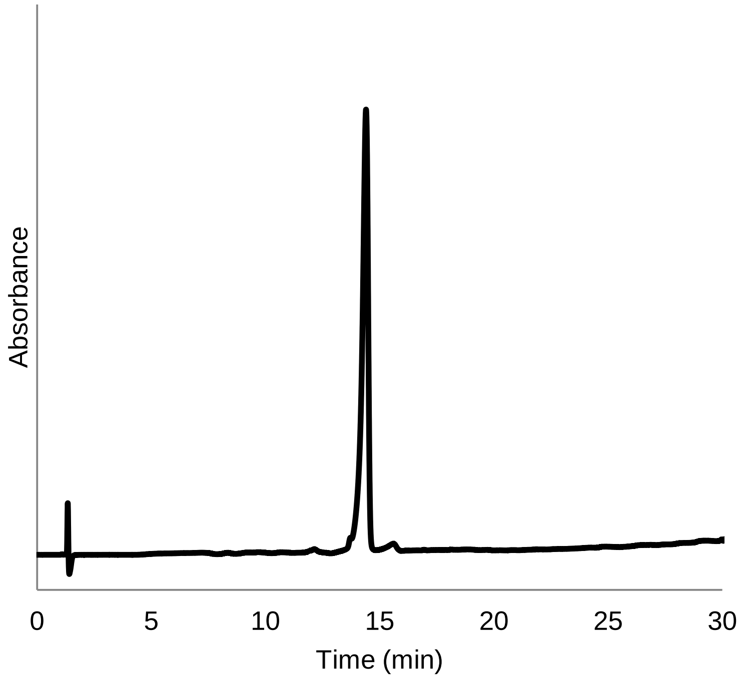

Analytical HPLC trace Peptoid 16 (*N*ae*N*spe*N*spe)$_4$

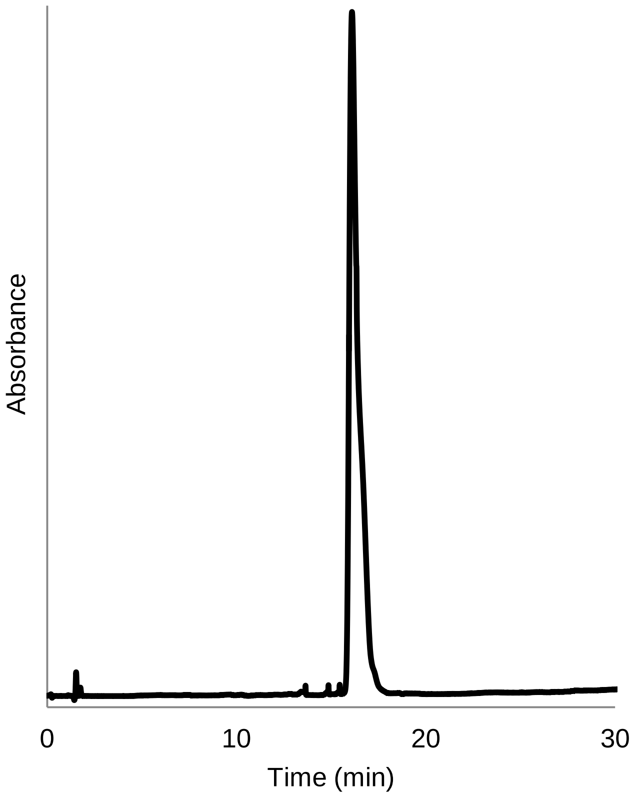

Figure S2. Analytical HPLC traces for representative peptoids in the library.

2.4. Chemical and Physical Data for Peptoid Library

Table S4. LC-MS data, RP-HPLC retention and the approximate yield after purification are tabulated for the peptoid library. Calculated and observed masses from LC-MS analysis of the peptoid library are shown. All peptoids are amidated at the C terminus. Typical HPLC gradient: 0 – 50% solvent B over 60 min then 50 – 100% solvent B over 25 mins (where solvent A = 95% H_2O, 5% MeCN, 0.1 % TFA; solvent B = 95% MeCN, 5% H_2O, 0.1% TFA), % MeCN calculated from middle of elution peak.

	Sequence	Mass Calculated *[M+H]+*	Mass Observed *$[M+H]^+$ or *$[M+2H]^{2+}$*	RP-HPLC Retention *(% MeCN)*
**1**	$(N\text{ah}N\text{phe}N\text{phe})_4$	1820.4	*910.6	38
**2**	$(N\text{ah}N\text{phe}N\text{phe})_3$	1369.8	*685.2	36
**3**	$(N\text{ah}N\text{phe}N\text{phe})_2$	918.2	*918.6	34
**4**	$(N\text{Lys}N\text{phe}N\text{phe})_4$	1707.2	1707.5	37
**5**	$(N\text{Lys}N\text{phe}N\text{phe})_3$	1284.6	1284.7	34
**6**	$(N\text{Lys}N\text{phe}N\text{phe})_2$	862.1	862.5	31
**7**	$(N\text{ae}N\text{phe}N\text{phe})_4$	1594.9	1595.2	37
**8**	$(N\text{ae}N\text{phe}N\text{phe})_3$	1201.5	1201.0	34
**9**	$(N\text{ae}N\text{phe}N\text{phe})_2$	806.0	806.4	30
**10**	$(N\text{ah}N\text{spe}N\text{spe})_4$	1931.2	*966.9	40
**11**	$(N\text{ah}N\text{spe}N\text{spe})_3$	1453.9	*727.1	40
**12**	$(N\text{ah}N\text{spe}N\text{spe})_2$	975.3	*974.6	38
**13**	$(N\text{Lys}N\text{spe}N\text{spe})_4$	1819.4	1819.4	42
**14**	$(N\text{Lys}N\text{spe}N\text{spe})_3$	1368.8	1368.8	37
**16**	$(N\text{Lys}N\text{spe}N\text{spe})_2$	918.2	918.6	33
**16**	$(N\text{ae}N\text{spe}N\text{spe})_4$	1708.2	1708.0	41
**17**	$(N\text{ae}N\text{spe}N\text{spe})_3$	1284.6	1284.7	39
**18**	$(N\text{ae}N\text{spe}N\text{spe})_2$	862.1	862.5	33

2.5. LC-MS Spectra for Peptoid Library

Peptoid 1 (*N*ah*N*phe*N*phe)$_4$

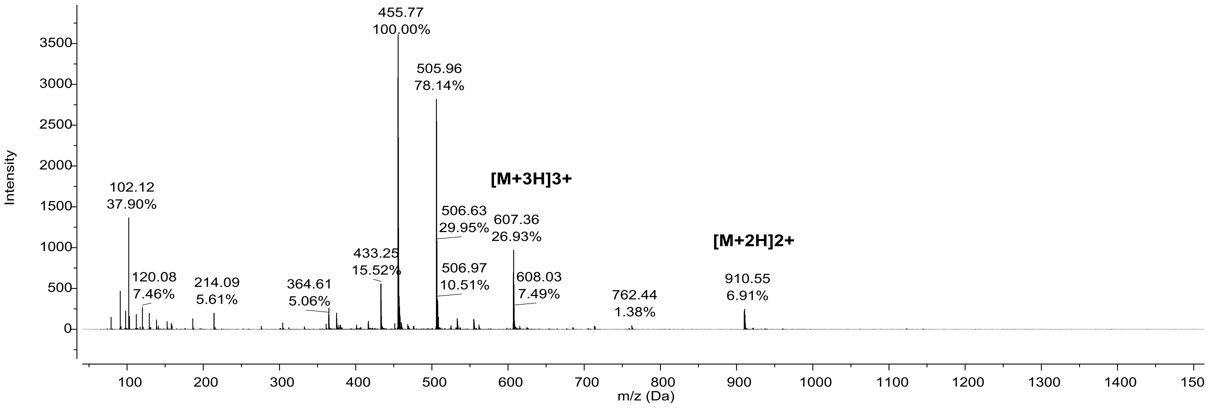

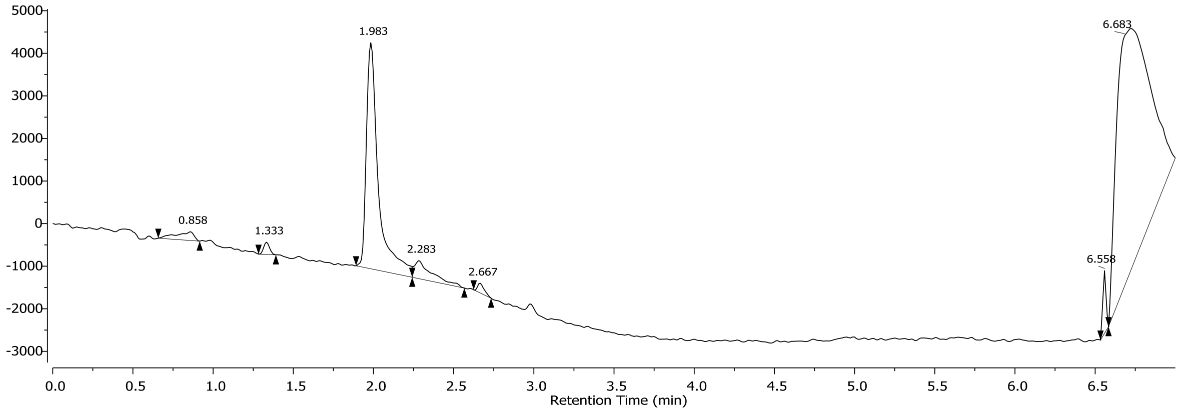

Peptoid 2 (*N*ah*N*phe*N*phe)$_3$

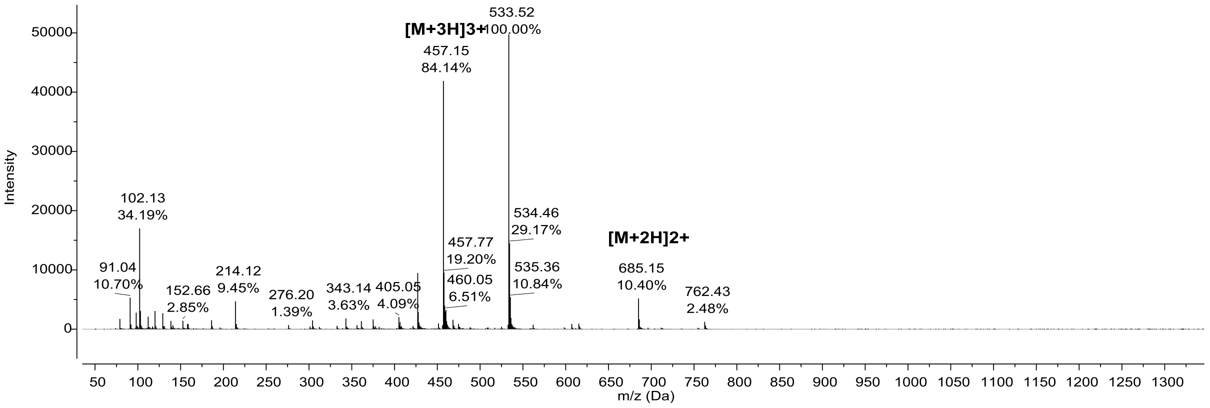

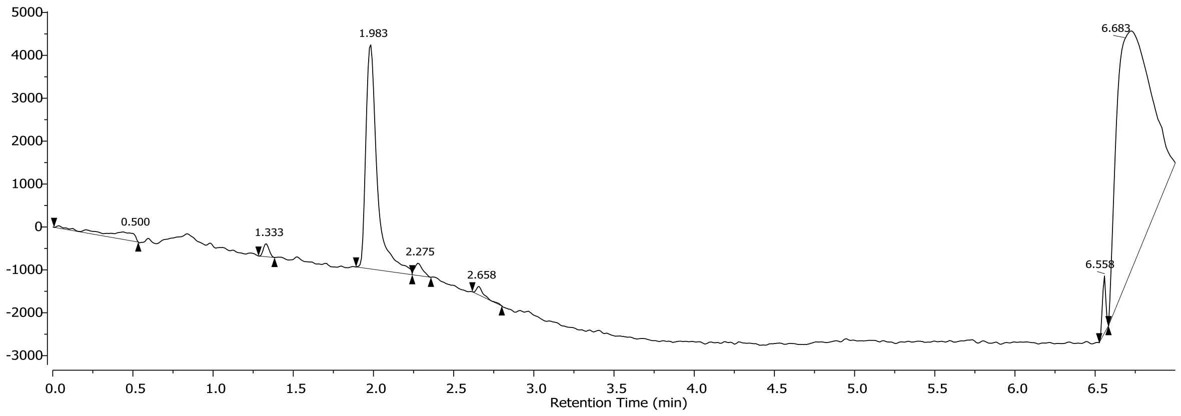

Peptoid 3 (*N*ah*N*phe*N*phe)$_2$

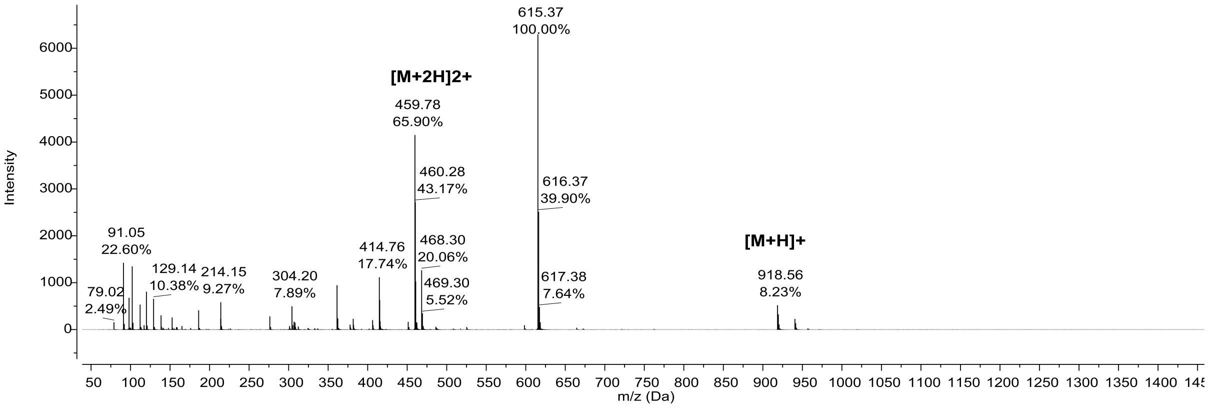

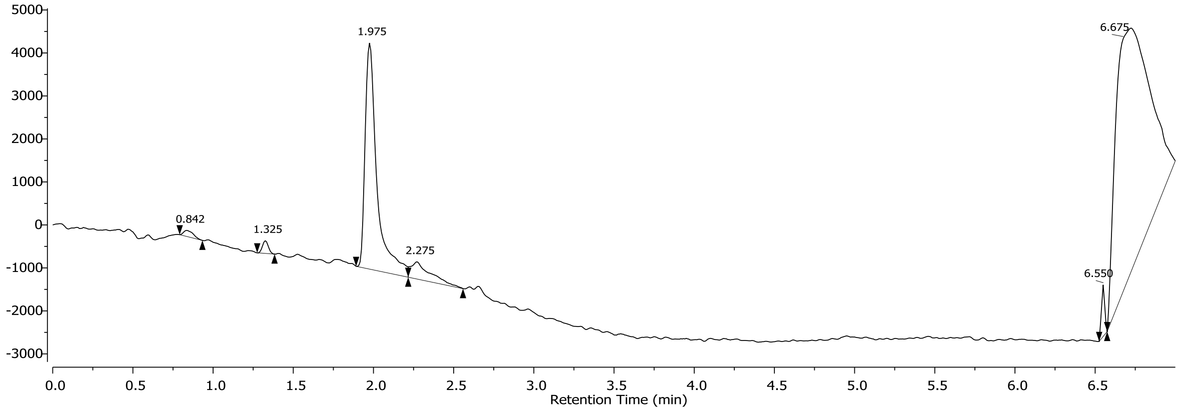

Peptoid 4 (*N*Lys*N*phe*N*phe)$_4$

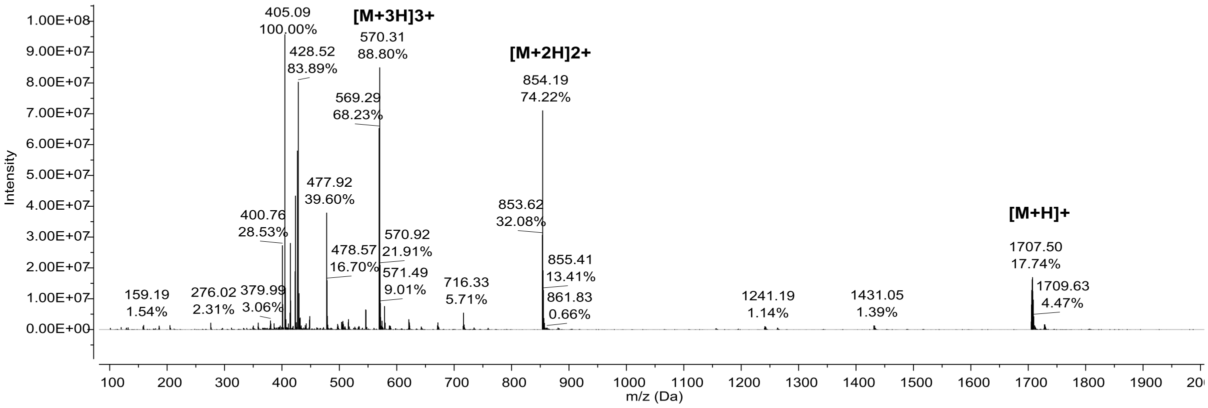

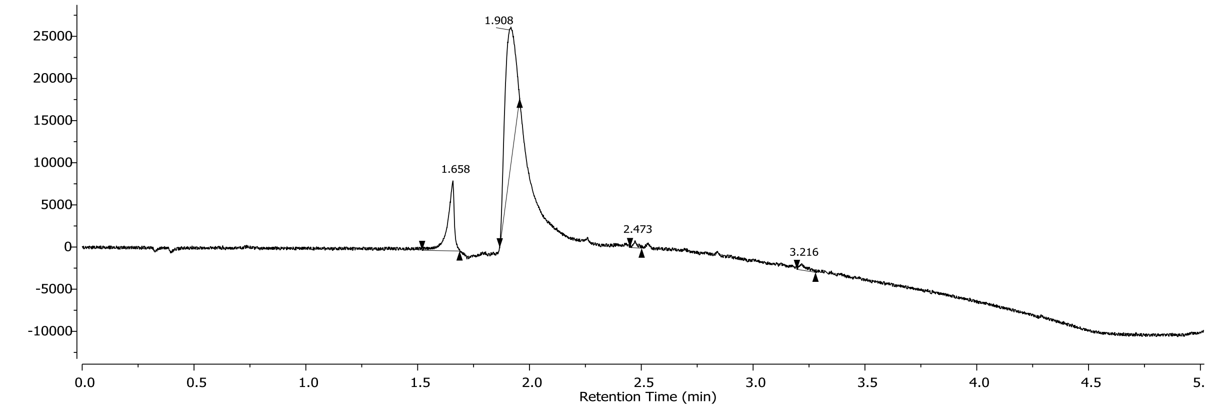

Peptoid 5 (*N*Lys*N*phe*N*phe)$_3$

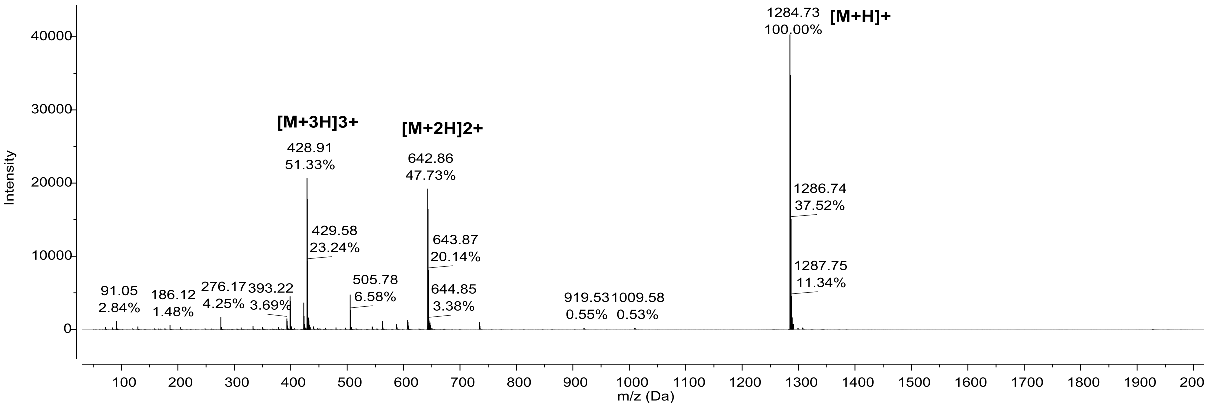

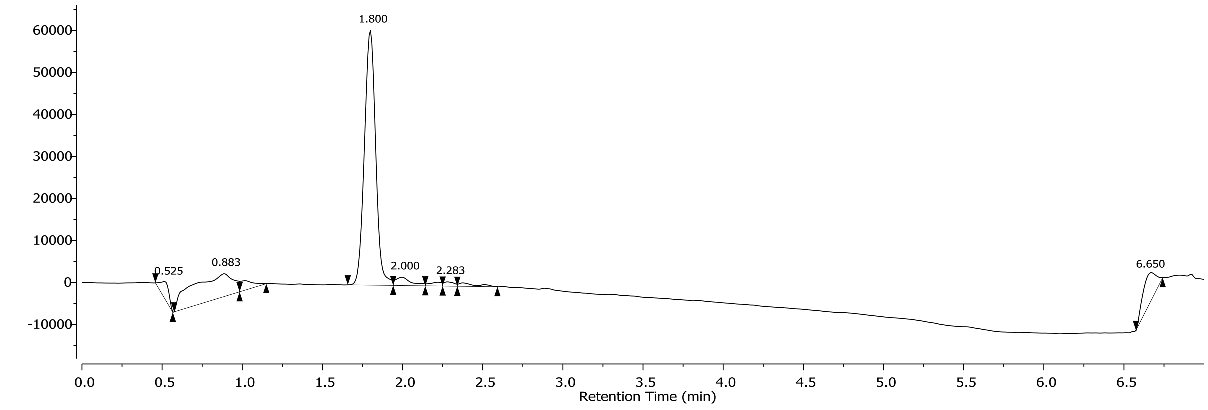

Peptoid 6 (*N*Lys*N*phe*N*phe)$_2$

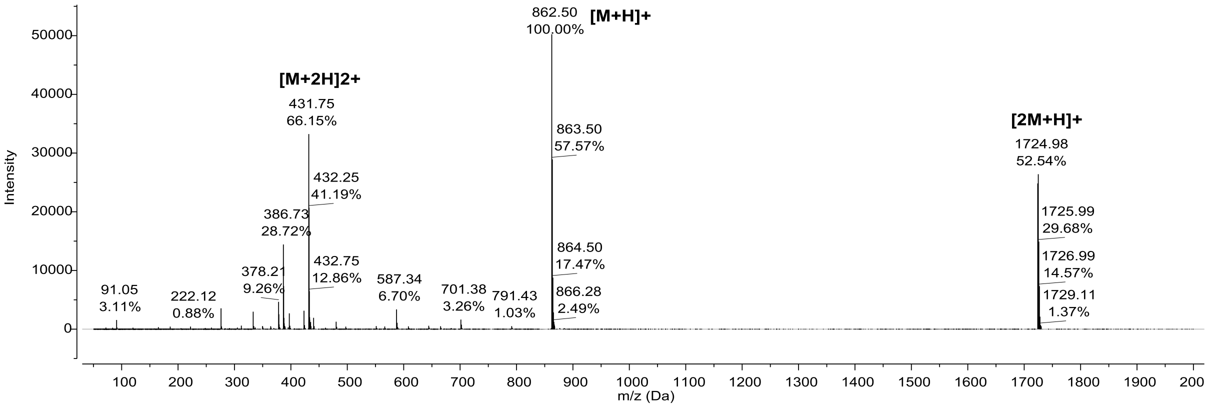

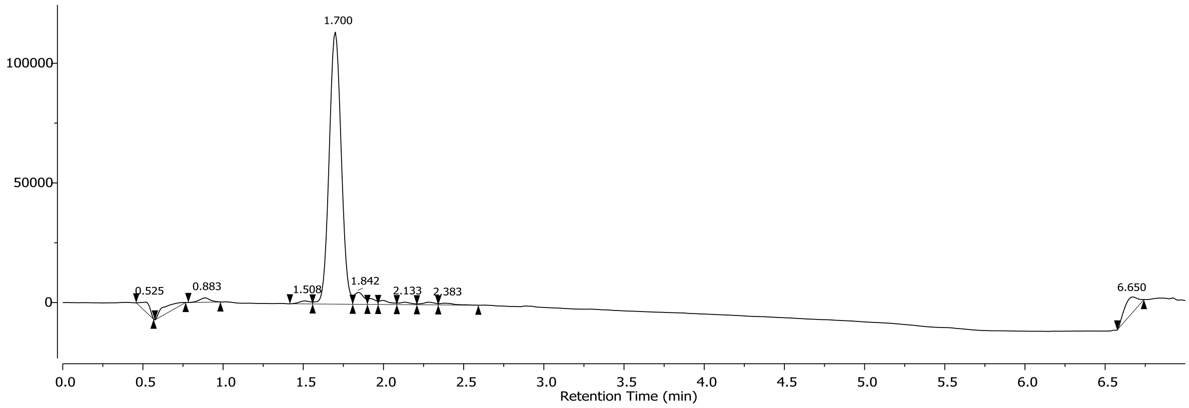

Peptoid 7 (*N*ae*N*phe*N*phe)$_4$

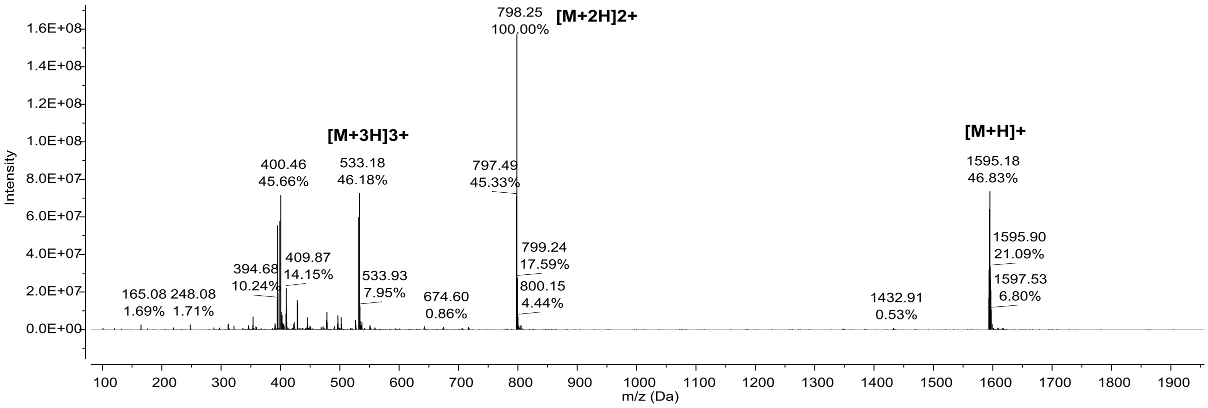

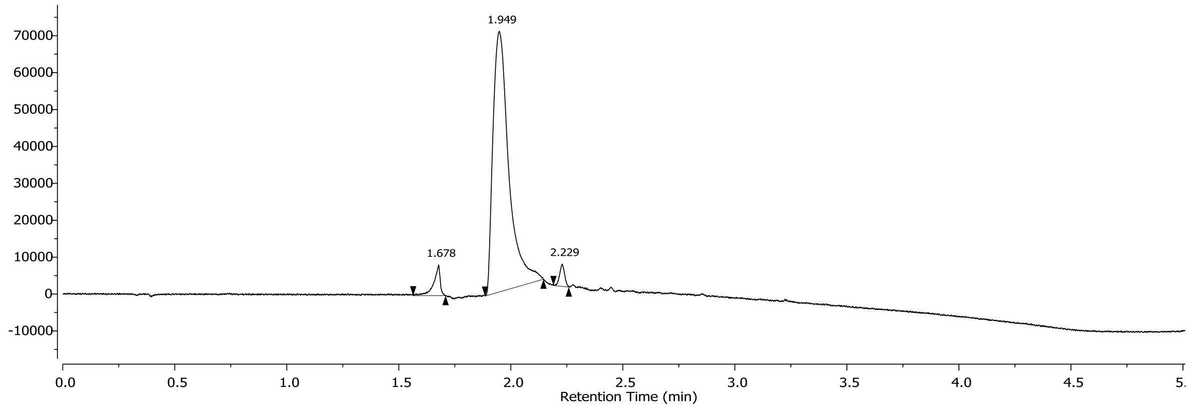

Peptoid 8 (*N*ae*N*phe*N*phe)$_3$

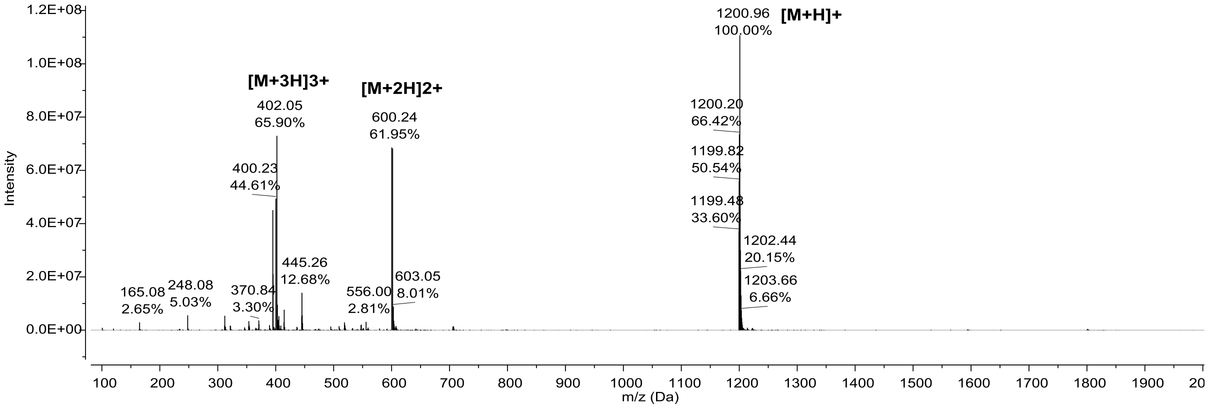

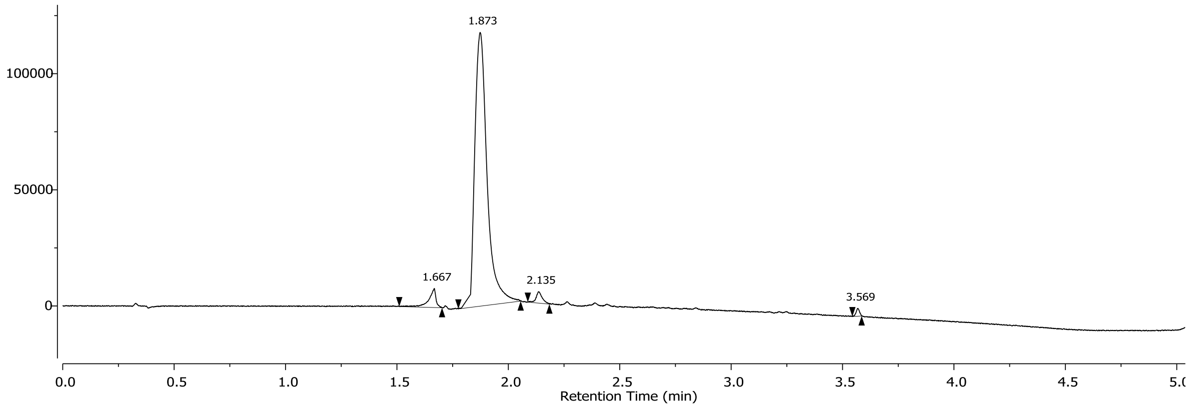

Peptoid 9 (*N*ae*N*phe*N*phe)$_2$

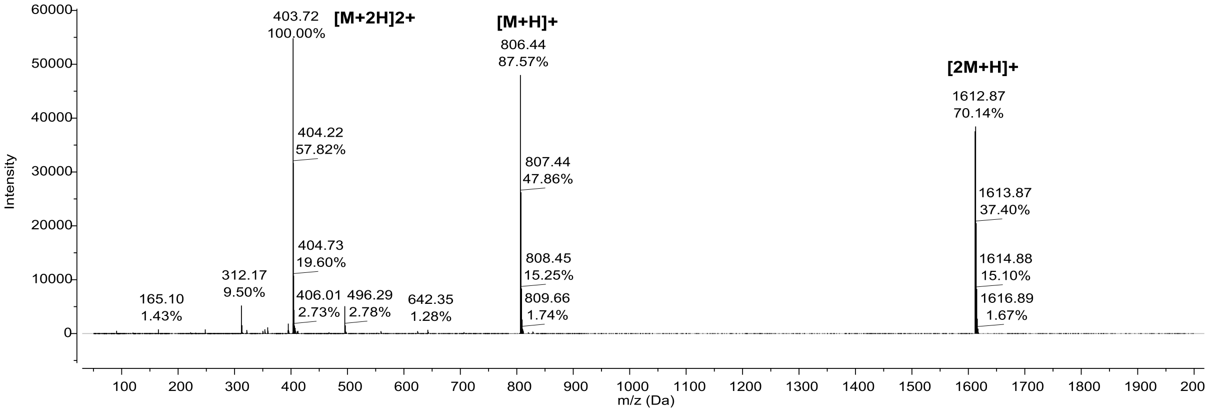

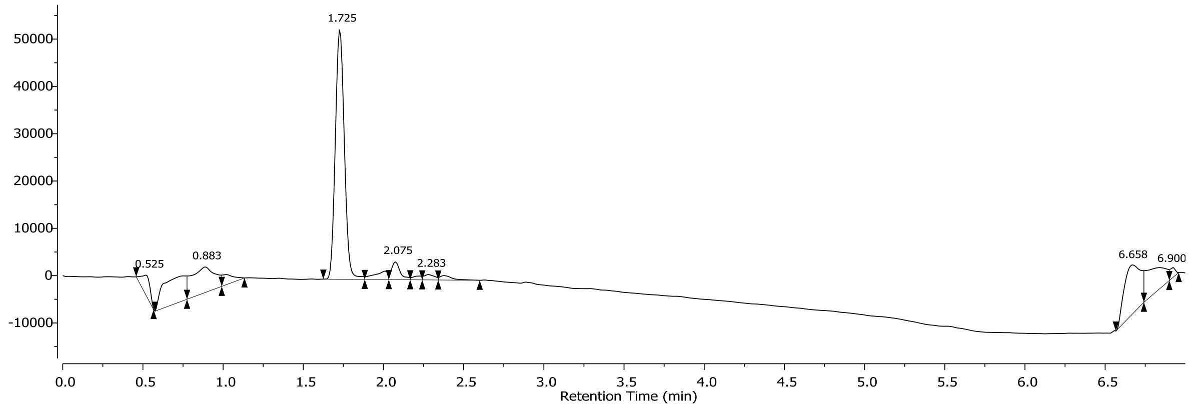

Peptoid 10 (*N*ah*N*spe*N*spe)$_4$

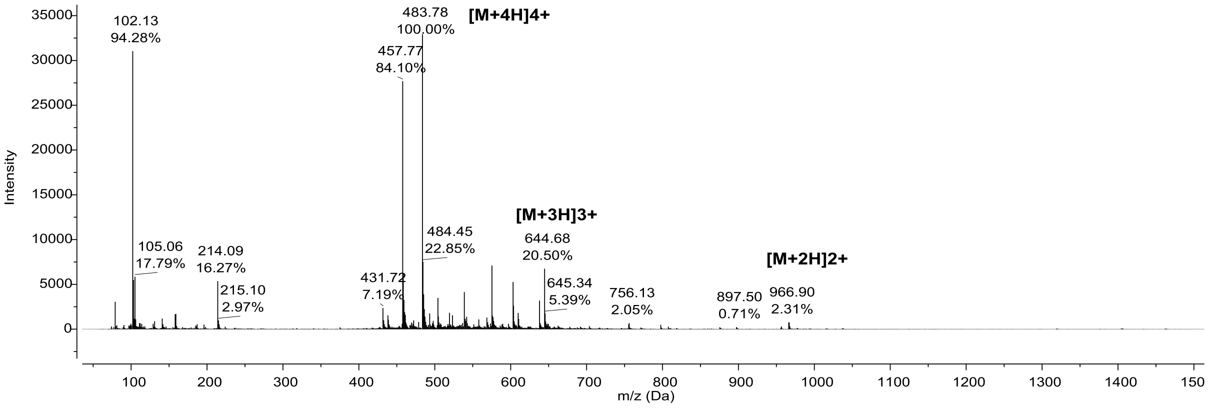

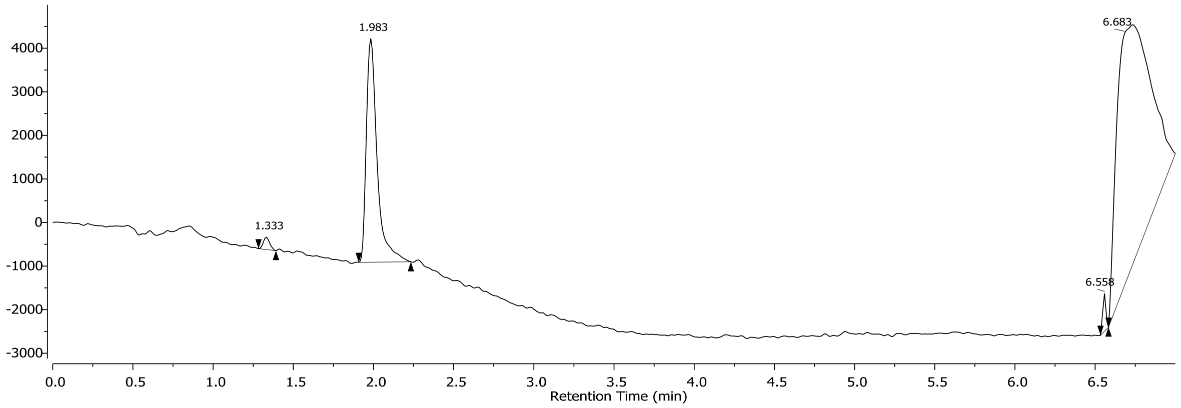

Peptoid 11 (*N*ah*N*spe*N*spe)$_3$

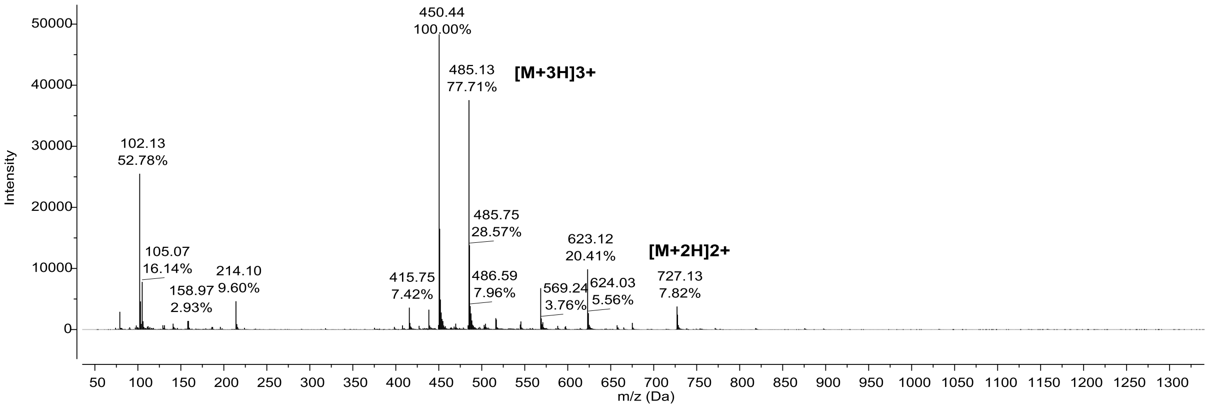

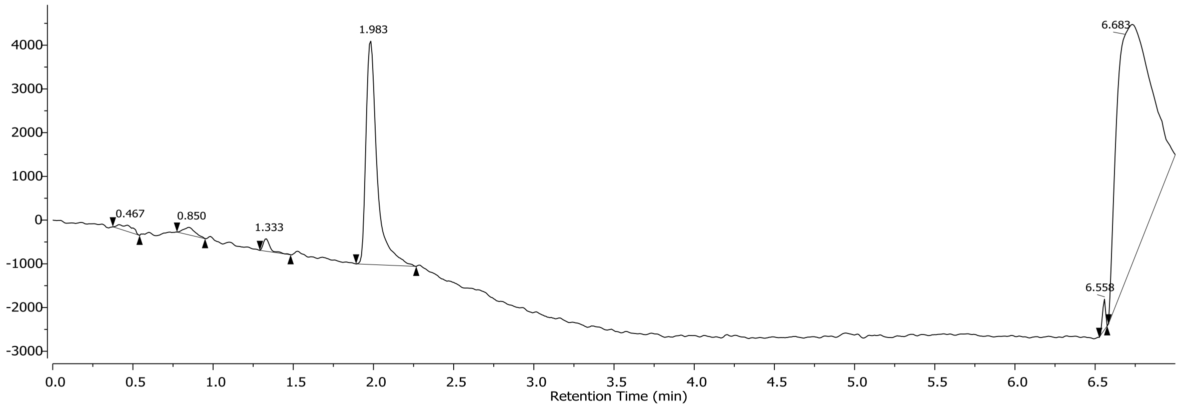

Peptoid 12 (*N*ah*N*spe*N*spe)$_2$

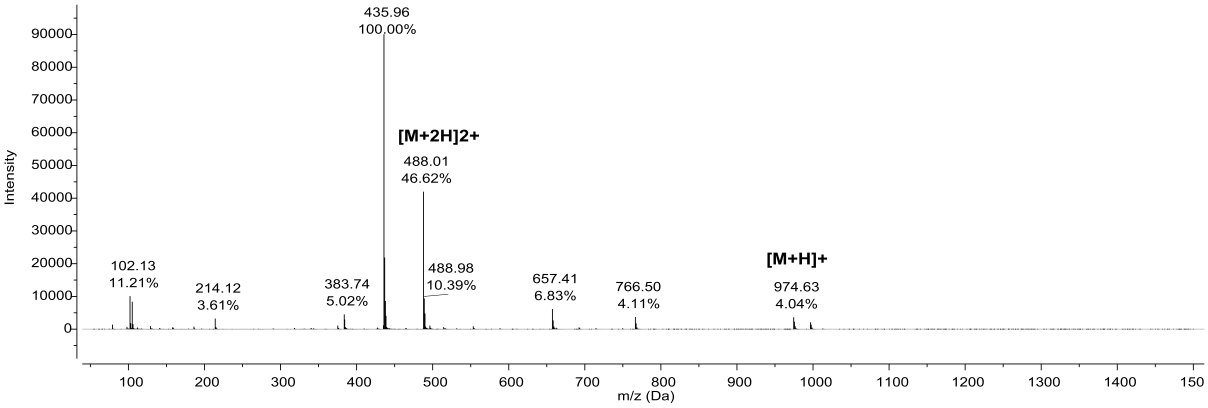

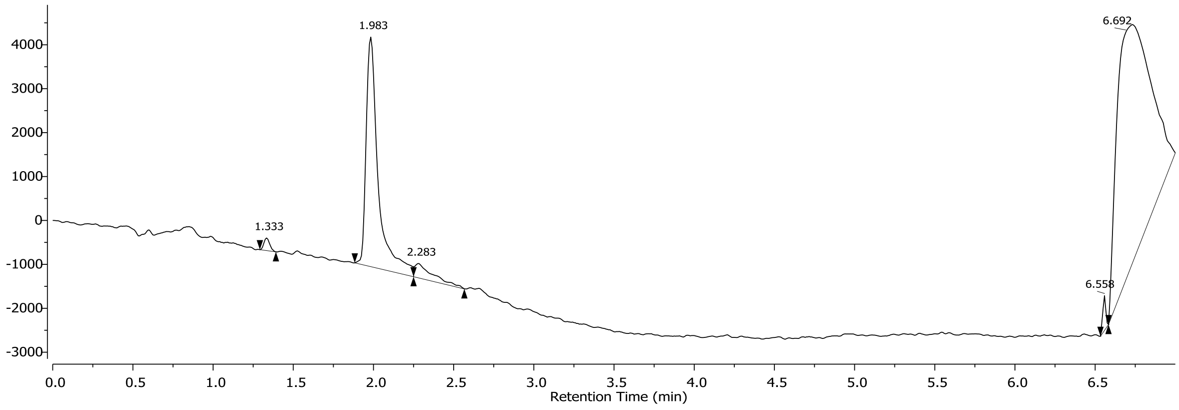

Peptoid 13 (*N*Lys*N*spe*N*spe)$_4$

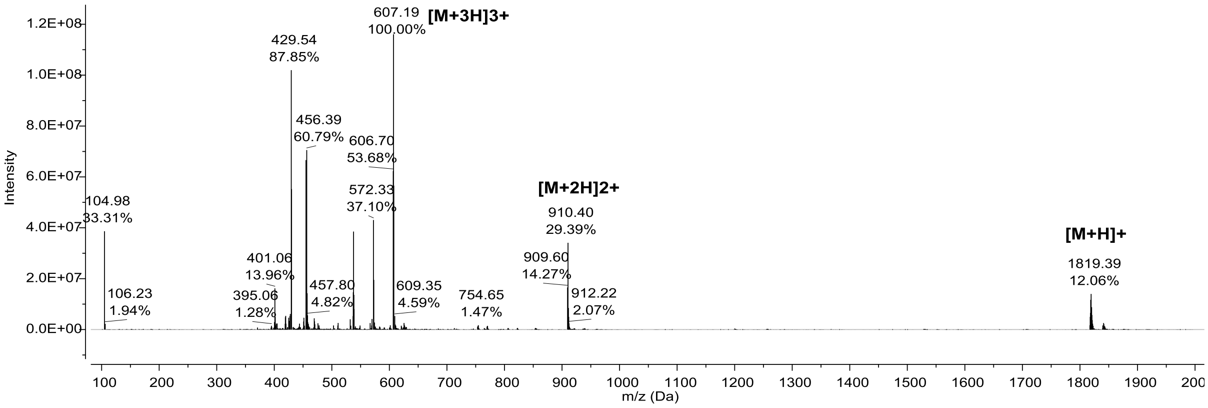

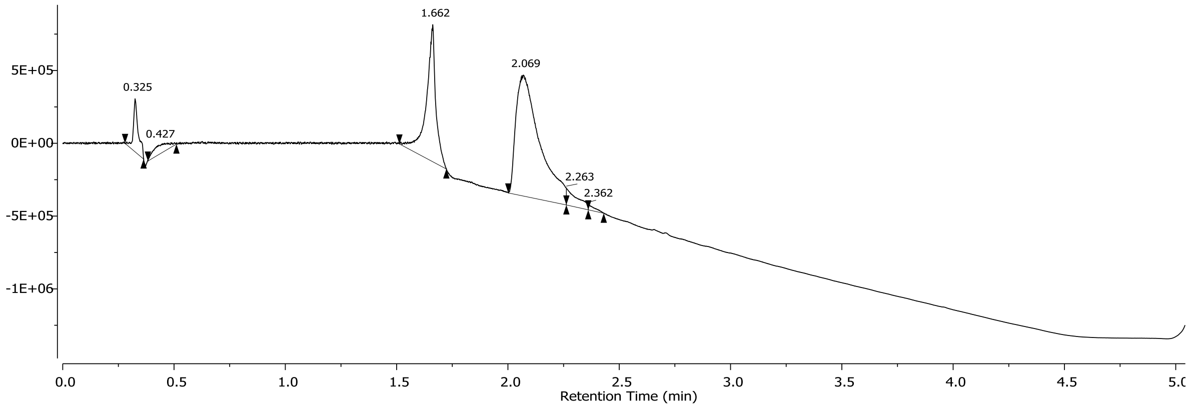

Peptoid 14 (*N*Lys*N*spe*N*spe)$_3$

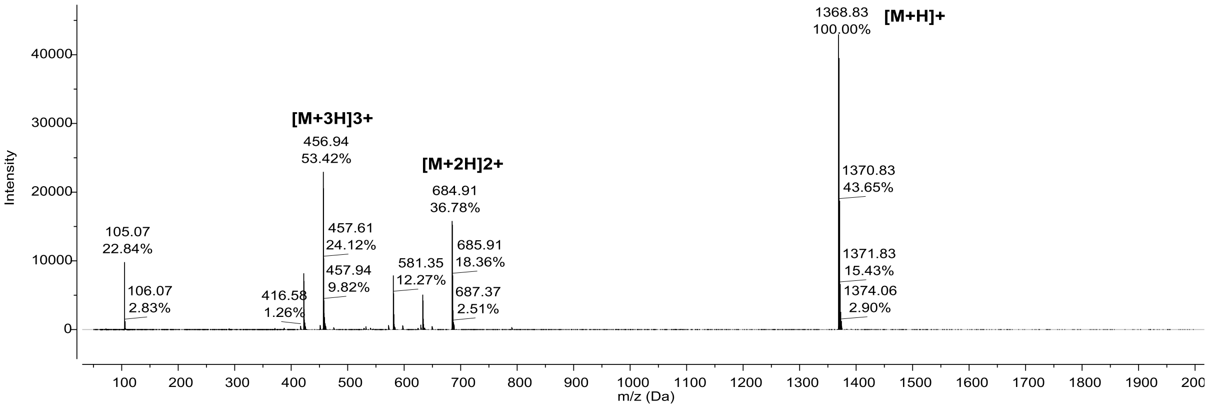

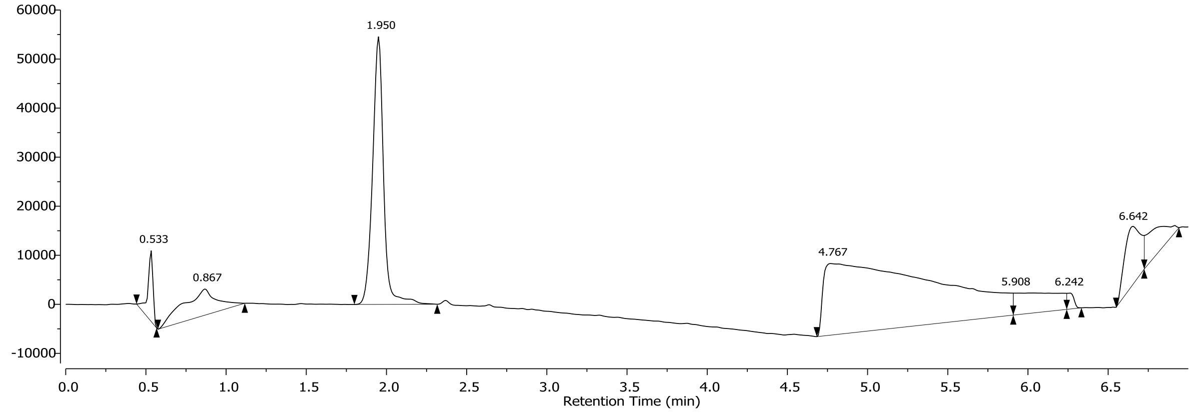

Peptoid 15 (*N*Lys*N*spe*N*spe)$_2$

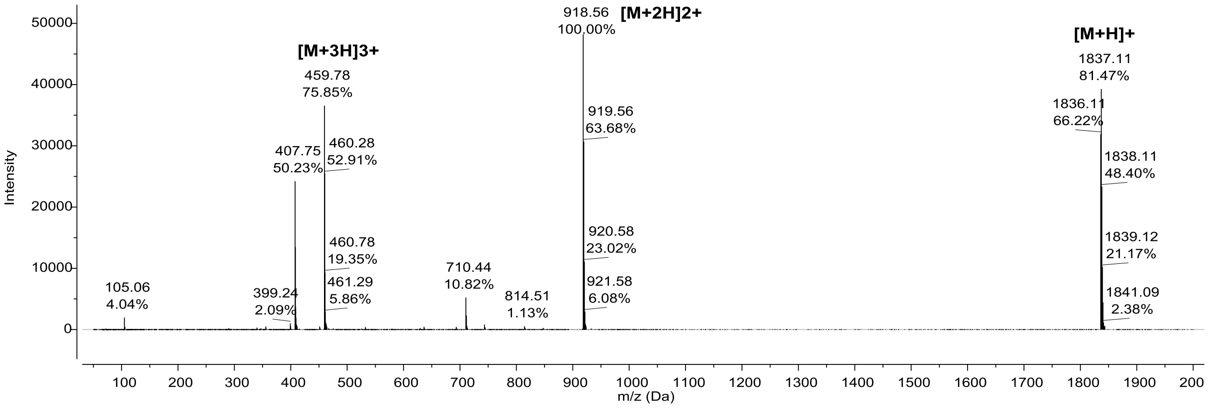

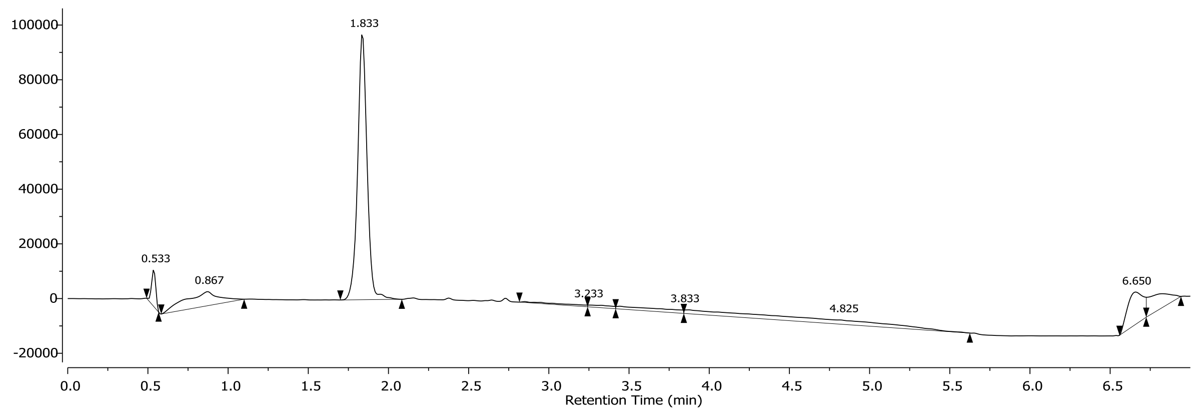

Peptoid 16 (*Nae**N*spe*N*spe)$_4$

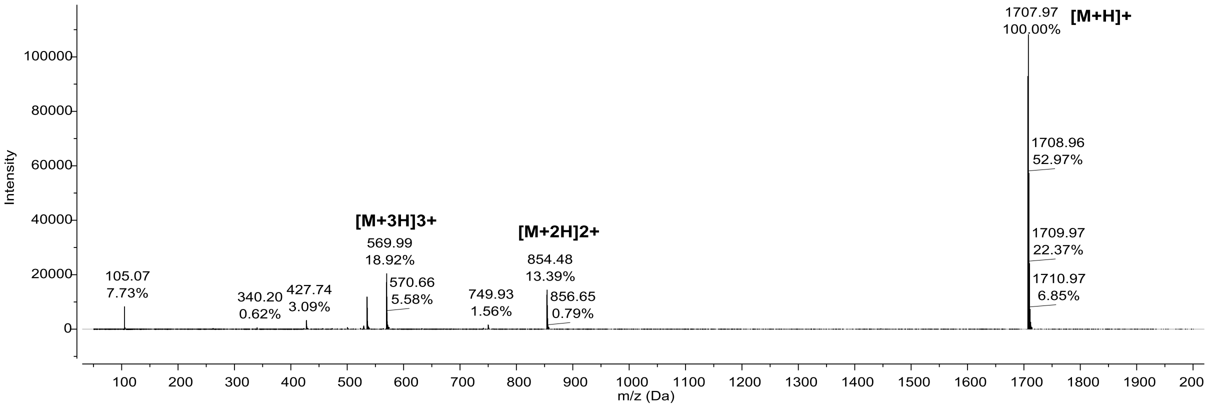

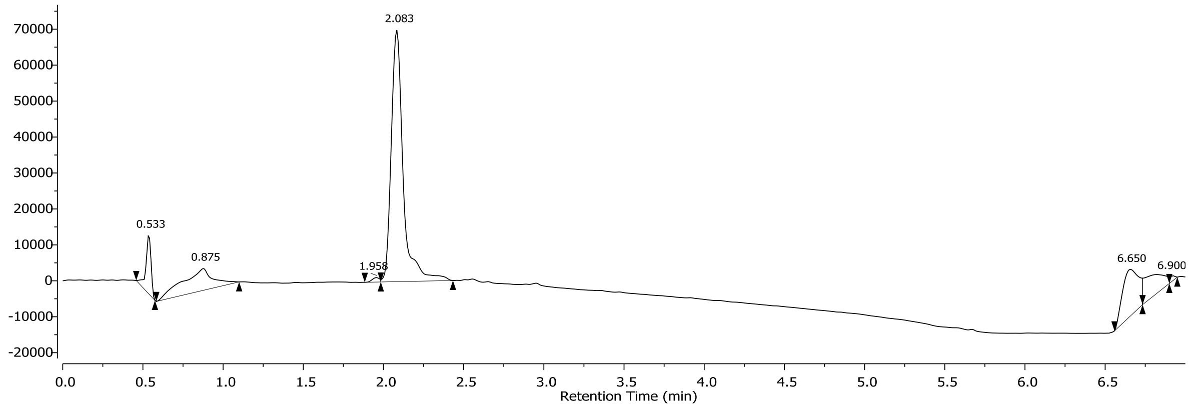

Peptoid 17 (*Nae**N*spe*N*spe)$_3$

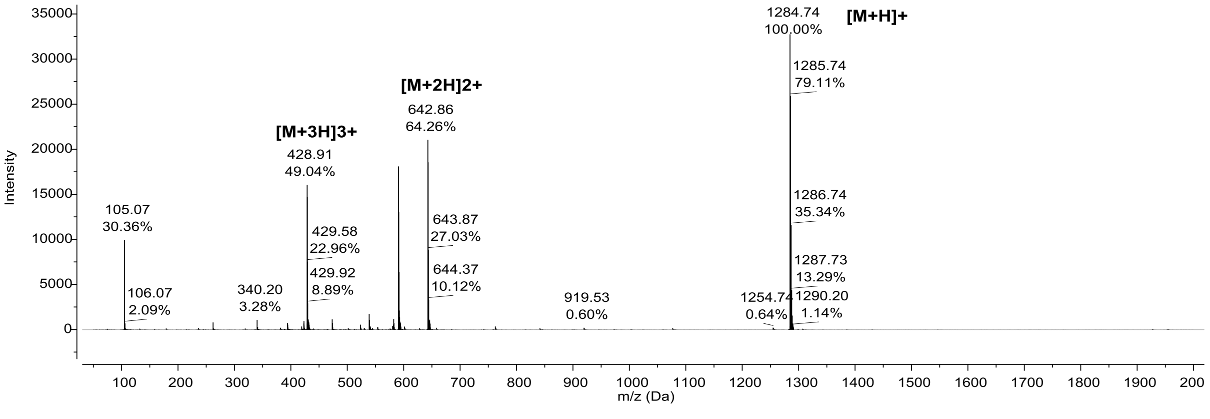

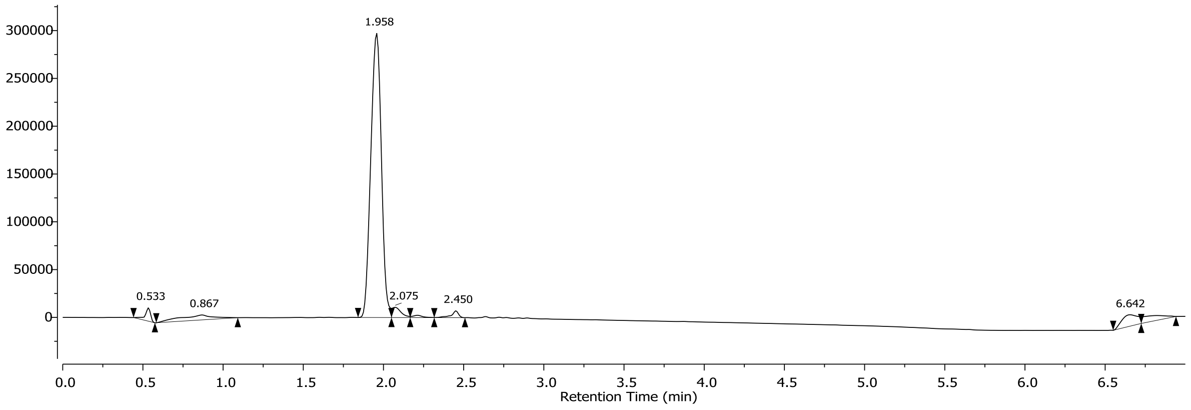

Peptoid 18 (*N*ae*N*spe*N*spe)$_2$

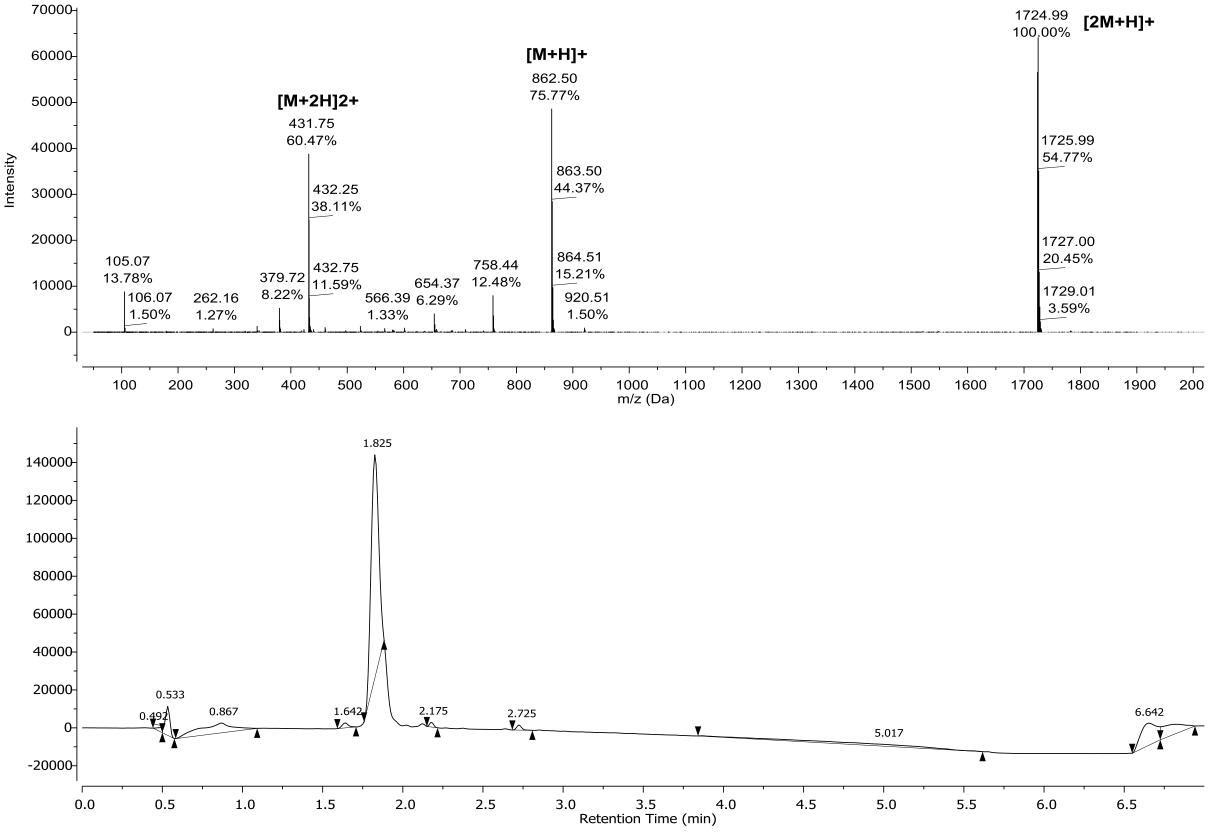

Figure S3. LC-MS spectra for the peptoid library; for each peptoid the mass spectrum (top) and UV chromatogram at λ = 250 nm (bottom) are shown.

3. qPCR reaction mixes and conditions

Supplementary Table S4. qPCR Reaction formulation for *C. albicans* (sequences from reference 42).

Component of reaction mix	Volume for 10 reactions	Final Concentration
FastStart Universal SYBR Green Master (2X) (Roche)	50 µl	1X
Forward primer (60 µM): CCTGTTTGAGCGTCRTTT	0.25 µl	150nM
Reverse primer (200 µM): TCCTCCGCTTATTGATAT	0.25 µl	500nM
Template	10 µl	
Nuclease free water	39.5 µl	

Supplementary Table S5. qPCR Reaction formulation for *S. aureus* (sequences from reference 43).

	Volume for 10 reactions	Final Concentration
Platinum® qPCR Supermix-UDG	60 µl	1X
Forward primer (200 µM): CAAAGCATCCTAAAAAAGGTGTAGAGA	0.24 µl	400 nM
Reverse primer (200 µM): TTCAATTTTCTTTGCATTTTCTACCA	0.24 µl	400 nM
Probe (100 µM): 6FAM-TTTTCGTAAATGCACTTGCTTCAGGACCA-BHQ1	0.24µl	200 nM
MgCl2 (50 mM)	2.4 µl	
Template	30 µl	
Nuclease free water	26.88 µl	

Supplementary Table S6. qPCR Reaction formulation for *E.coli* (sequences from reference 44).

	Volume for 10 reactions	Final Concentration
FastStart Universal SYBR Green Master (2X) (Roche)	50 µl	1X
Forward primer (200 µM): AGAAGCTTGCTCTTTGCTGA	0.25 µl	500nM
Reverse primer (200 µM): CTTTGGTCTTGCGACGTTAT	0.25 µl	500nM
Template	10 µl	
Nuclease free water	39.5 µl	

Supplementary Table S7. qPCR Conditions for *C. albicans* and *E. coli* (instructions provided with FastSart kit, Roche).

Cycles	Target temperature	Hold time	Analysis Mode
**1**	50ºC	15 min	None
**1**	95ºC	10 min	None
**45**	95ºC	15 s	None
	60ºC	60 s	Single fluorescence acquisition
**Melting analysis**			
**1**	95ºC	30 s	None
**1**	35ºC	60 s	None
**1**	98ºC	30 s	Continuous fluorescence acquisition
**Cooling**			
**1**	40ºC	10 s	None

Supplementary Table S8. qPCR Conditions for *S. aureus* (adapted from instructions provided with Platinum® Quantitative PCR SuperMix-UDG).

Cycles	Target temperature	Hold time	Analysis Mode
**1**	50ºC	15 min	None
**1**	95ºC	5 min	None
**45**	95ºC	10 s	None
	60ºC	60 s	Single fluorescence acquisition
**Cooling**			
**1**	40ºC	10 s	None

4. Proteolytic Stability Study

Although it is generally acknowledged that peptoid backbone structures should be inherently resistant proteolysis, we compared the tryptic digestion profile of one selected peptoid, peptoid **7**, against the naturally occurring alpha helical peptide LL-37. Peptoid **7** showed no degradation following treatment with trypsin for 24 hours whereas LL-37 was completely degraded to peptide fragments during the same time period.

A

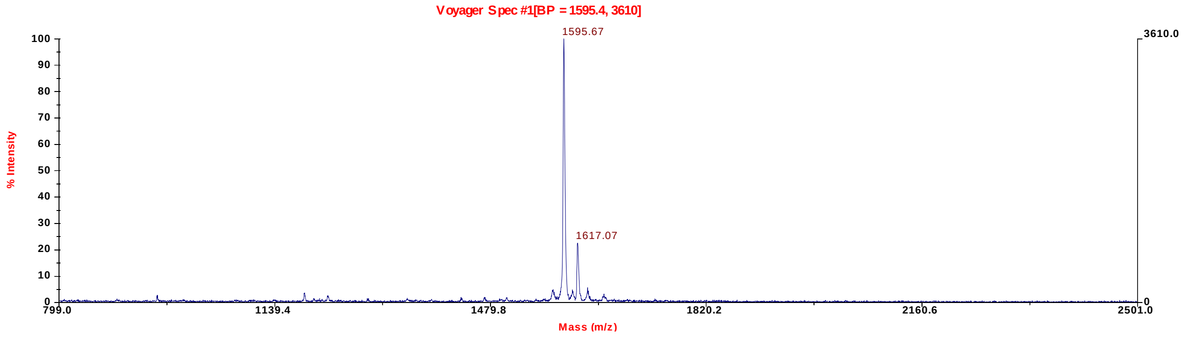

B

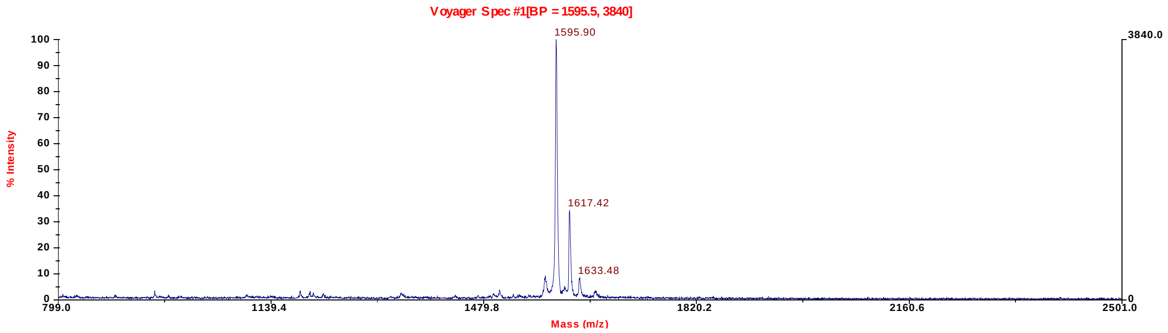

C

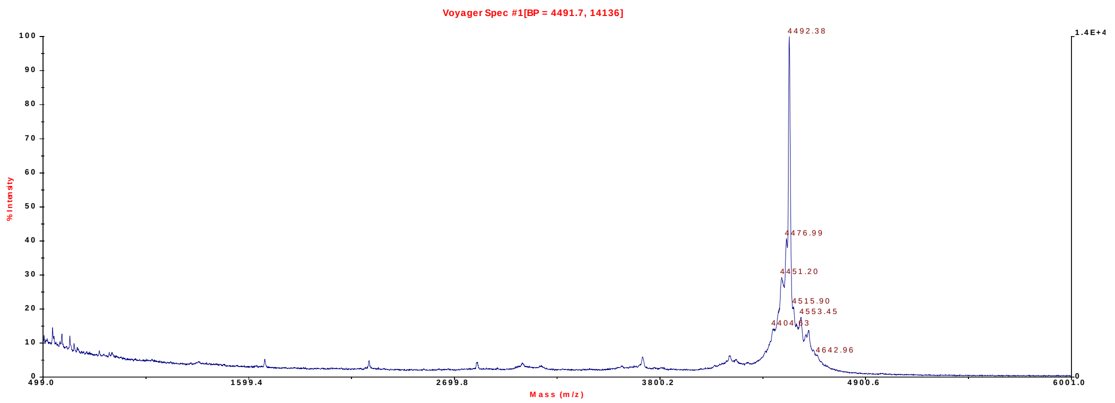

D

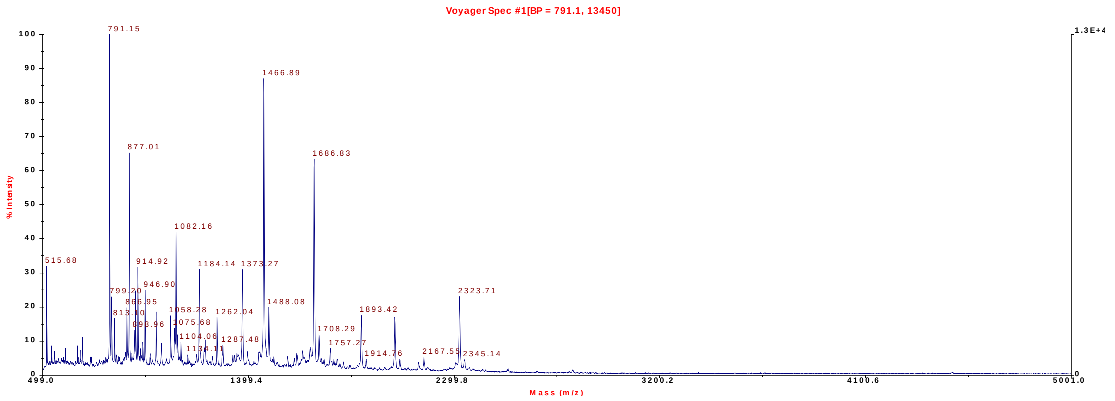

Tryptic degradation profiles of peptoid 7 (A, B) versus LL-37 (D, E). Peptoid 7 at T=0 hrs (A) and T= 24 hrs (B), showing no degradation following treatment with trypsin for 24 hrs. LL-37 at T=0 (C) and T=24 hrs (D), showing complete loss of the parent molecule and its replacement by lower molecular mass peptide fragments after 24 hours.

5. Toxicity to mammalian cell lines

Toxicity was determined using an alamarBlue® cytotoxicity assay. The majority of the peptoids had ED_{50} values > 100 μM (Table 2), indicating that they had minimal cytotoxic effects on model human endothelial (HepG2) and keratinocyte (HaCaT) cell lines. The only sequences that showed toxicity were the longest 12 residue peptoids containing the chiral *N*spe monomer (Table 2). Peptoids **5**, **7** and **17**, which were selected for further study, had ED_{50} values > 100 μM (Table 2).

Peptoid	Sequence	ED_{50} (μM)	
		**HaCaT**	**HepG2**
**1**	(*N*Lys*N*phe*N*phe)$_4$	36 ± 1	>100
**2**	(*N*Lys*N*phe*N*phe)$_3$	>100	>100
**3**	(*N*Lys*N*phe*N*phe)$_2$	>100	>100
**4**	(*N*Lys*N*spe*N*spe)$_4$	20 ± 1	29 ± 1
**5**	(*N*Lys*N*spe*N*spe)$_3$	>100	>100
**6**	(*N*Lys*N*spe*N*spe)$_2$	>100	>100
**7**	(*N*ae*N*phe*N*phe)$_4$	>100	>100
**8**	(*N*ae*N*phe*N*phe)$_3$	>100	>100
**9**	(*N*ae*N*phe*N*phe)$_2$	>100	>100
**10**	(*N*ae*N*spe*N*spe)$_4$	26 ± 2	41 ± 2
**11**	(*N*ae*N*spe*N*spe)$_3$	>100	>100
**12**	(*N*ae*N*spe*N*spe)$_2$	>100	>100
**13**	(*N*ah*N*phe*N*phe)$_4$	>100	>100
**14**	(*N*ah*N*phe*N*phe)$_3$	>100	>100
**15**	(*N*ah*N*phe*N*phe)$_2$	>100	>100
**16**	(*N*ah*N*spe*N*spe)$_4$	23 ± 1	41 ± 1
**17**	(*N*ah*N*spe*N*spe)$_3$	>100	>100
**18**	(*N*ah*N*spe*N*spe)$_2$	>100	>100
